# HMGCL-induced β-hydroxybutyrate production attenuates hepatocellular carcinoma via DPP4-mediated ferroptosis susceptibility

**DOI:** 10.1007/s12072-022-10459-9

**Published:** 2022-12-12

**Authors:** Xiaohan Cui, Xiao Yun, Meiling Sun, Renzhi Li, Xiajie Lyu, Yuanxiang Lao, Xihu Qin, Wenbin Yu

**Affiliations:** 1 Department of Gastrointestinal Surgery, General Surgery, Qilu Hospital, Cheeloo College of Medicine, Shandong University, Jinan, 250012 Shandong People’s Republic of China; 2 Nanjing Medical University, Nanjing, 211166 Jiangsu People’s Republic of China; 3 Department of General Surgery, The Affiliated Changzhou No. 2 People’s Hospital of Nanjing Medical University, 68 Pohu Middle Road, Changzhou, 213000 Jiangsu People’s Republic of China; 4 Department of Hepatobiliary Surgery, The Affiliated Drum Tower Hospital of Nanjing University Medical School, 321 Zhongshan Road, Nanjing, 210008 Jiangsu People’s Republic of China; 5 Gangarosa Department of Environmental Health, Rollins School of Public Health, Emory University, Atlanta, GA 30332 USA

**Keywords:** HMGCL, Hepatocellular carcinoma, Ferroptosis, Acetylation, DPP4

## Abstract

**Background:**

Metabolic disorder is an essential characteristic of tumor development. Ketogenesis is a heterogeneous factor in multiple cancers, but the effect of ketogenesis on hepatocellular carcinoma (HCC) is elusive.

**Methods:**

We aimed to explain the role of ketogenesis-related hydroxy-methyl-glutaryl-CoA lyase (HMGCL) on HCC suppression. Expression pattern of HMGCL in HCC specimens was evaluated by immunohistochemistry (IHC). HMGCL was depleted or overexpressed in HCC cells to investigate the functions of HMGCL in vitro and in vivo. The anti-tumor function of HMGCL was studied in subcutaneous xenograft and *Trp53*^*Δhep/Δhep*^*; c-Myc*-driven HCC mouse models. The mechanism of HMGCL-mediated tumor suppression was studied by IHC, western blot (WB) and Cut & Tag.

**Results:**

HMGCL depletion promoted HCC proliferation and metastasis, whereas its overexpression reversed this trend. As HMGCL catalyzes β-hydroxy-butyric acid (β-OHB) production, we discovered that HMGCL increased acetylation at histone H3K9, which further promoted the transcription of dipeptidyl peptidase 4 (DPP4), a key protein maintains intracellular lipid peroxidation and iron accumulation, leading to HCC cells vulnerability to erastin- and sorafenib-induced ferroptosis.

**Conclusion:**

Our study identified a critical role of HMGCL on HCC suppression, of which HMGCL regulated H3K9 acetylation through β-OHB and modulating the expression of DPP4 in a dose-dependent manner, which led to ferroptosis in HCC cells.

**Supplementary Information:**

The online version contains supplementary material available at 10.1007/s12072-022-10459-9.

## Introduction

The high mortality and recurrence rates of HCC make it the second leading factor in cancer-related deaths worldwide [1–3]. Current therapeutic strategies of HCC include surgical resection, interventional or radiofrequency ablation, chemotherapy or targeted therapy and liver transplantation [4], of which the overall survival (OS) remains unsatisfactory [5, 6]. Therefore, there is an urgent need to investigate the new diagnostic and therapeutic targets for HCC.

Metabolic reprogramming is a well-established tumor characteristic that appears in cancers to meet the needs for biosynthesis, rapid proliferation and metastasis [7–9]. As described previously, ketone bodies act as metabolic products and energy suppliers during starvation [10], which is involved in the pathological state of variety of tumors. In HCC and prostate cancer, ketogenesis-related hydroxy-methyl-glutaryl-CoA synthase 2 (HMGCS2) inhibits tumor proliferation and metastasis [11–13], whereas exogenous ketone bodies also show the similar effects in pancreatic cancer [14, 15]. In addition, ketogenesis also plays a role in epigenetic modifications that affect the acetylation levels of several histones, which is essential for gene expression, chromatin remodeling, transcription factor activity, etc. [10]. Ketogenesis-related HMGCL has heterogeneous effects on multiple tumors. In melanoma, HMGCL is upregulated by BRAF through OCT1 activation, stimulating the MEK-ERK pathway via an acetoacetic acid-dependent manner [16]. HMGCL is also one of the most significantly upregulated genes in pancreatic cancer, which contributes to produce β-OHB and provides additional energy for proliferation and metastasis of pancreatic cancer [17]. In contrast, HMGCL expression in nasopharyngeal carcinoma promotes the production of reactive oxygen species (ROS) and inhibiting nasopharyngeal carcinoma cell development and metastasis [18]. However, the mechanism of HMGCL inhibited proliferation and metastasis in HCC is still poorly excavated.

Recently, several forms of cell death have been classified and proven to be either accidental or controlled cell death [19, 20]. In contrast to accidental cell death, regulated (active) cell death (RCD) is stimulated by a range of signaling pathways [19, 20]. Ferroptosis is a type of non-apoptotic cell death defined as an iron-dependent RCD caused by excessive lipid peroxidation-mediated membrane damage [21, 22], the toxicity of which has been reported as an evolutionarily conserved program and plays a role on the development of multiple diseases in eukaryotes [23, 24]. Although the term “ferroptosis” was coined in 2012, the initial theoretical view of iron toxicity may have developed from the following aspects of nutrient (particularly cysteine) depletion-induced cancer cell death and “oxidation” [21]. Recently, it was reported that a variety of pharmacological or natural compounds, as well as cell-intrinsic proteins, regulate ferroptosis [25]. Therefore, novel ferroptosis-related biomarkers have glowing prospective.

In this study, we identified HMGCL as a suppressor in HCC proliferation and metastasis. HMGCL is involved in cell death process in HCC cells via regulating β-OHB production and commitment histone H3K9 acetylation to mediate the expression of ferroptosis-related DPP4, leading to enhanced HCC cell death. Our study paves a novel way to serve HMGCL as a therapeutic target against HCC proliferation and metastasis.

## Materials and methods

### Human liver samples

Human liver tissue samples were collected from 332 HCC patients in two independent cohorts. HCC tissues and NLT collected from 252 HCC patients who underwent hepatectomy at Zhongshan Hospital of Fudan University were defined as cohort 1. Liver tissue samples from cohort 2 were collected from 80 HCC patients who underwent hepatectomy at Nanjing Drum Tower Hospital. This study was approved by the Institutional Ethics Committee of Nanjing Drum Tower Hospital and Zhongshan Hospital in accordance with the 1975 Declaration of Helsinki. All patients signed written informed consent for tissue analysis prior to the procedure. The clinical and pathological characteristics of patients with HCC at Zhongshan Hospital, Fudan University, are listed in Table 1.

**Table 1:** Univariate and Multivariate Cox regression analysis for OS and RR in 252 HCC patients

Variables	Overall survival				Recurrence rate			
	Unvariate	Multivariate			Unvariate	Multivariate		
	*p* value	Hazard ratio	95% CI	*p* value	*p* value	Hazard ratio	95% CI	*p* value
HMGCL: high vs. low	0.0456	0.664	0.458–0.906	0.012	0.0161	0.742	0.555–0.993	0.045
Age: < 51 vs. 51 year	0.1469			NA	0.757			NA
Gender: female vs. male	0.145			NA	0.809			NA
Liver cirrhosis: no vs. yes	0.725			NA	0.336			NA
Hepatitis Bs antigen: no vs. yes	0.875			NA	0.661			NA
α-Fetoprotein: < 20 vs. ≥ 20 ng/ml	< 0.001			NA	0.002			NA
Tumor size: < 5 vs. ≥ 5 cm	< 0.001	1.247	1.206–1.288	< 0.001	< 0.001	1.224	1.185–1.264	< 0.001
Tumor encapsulation: complete vs. incomplete	0.052			NA	0.001			NA
Microvascular invasion: no vs. yes	< 0.001	2.2537	2.160–2.979	< 0.001	< 0.001	2.389	2.040–2.799	< 0.001
Intrahepatic metastasis: no vs. yes	NA	4.96	3.732–6.592	< 0.001	NA	3.558	2.820–4.488	< 0.001
Tumor differentiation: low vs. high	0.001			NA	0.007			NA
pTNM stage: I & II vs. III	< 0.001			NA	< 0.001			NA

*NA：* not adapted, *NS：* no significant

### Animal studies

Mice are kept under specific pathogen-free and temperature-controlled conditions with 12 h of diurnal alternation at 22–24 °C. Nude mice were obtained from Shanghai Southern Model Biotechnology Co. *Trp53*^*flox/flox*^ mice were a kind gift from Southern Medical University. *Trp53*^*flox/flox*^ mice were crossed with Alb-Cre mice to generate hepatocyte-specific *Trp53* knockout (*Trp53*^*Δhep/Δhep*^) mice. Mixture used for hydrodynamic tail vein injection included a sterile 0.9% NaCl solution/plasmid mix containing 5 μg of pSB-U6-sgHMGCL-CBH-Cas9 or 5 μg of pSB-EF1A-FLAG-HMGCL, 5 μg of pT3-MYC and 2.5 μg of CMV-SB13 transposase. A total volume of mixture corresponding to 10% of body weight was injected via the tail vein in 7 s into 8-week-old male *Trp53*^Δhep/Δhep^ mice. After 6 weeks, mice were humanely sacrificed by CO_2_ asphyxiation. For the subcutaneously implanted tumor model in nude mice, 3 × 10^6^ Huh7 or MHCC-LM3 cells, were harvested and re-suspended in 100 μl of PBS and injected subcutaneously into 4–6-week-old male BALB/c nude mice. Tumor volumes were measured as (tumor width^2^ × tumor length)/2 and recorded every 4 days. Finally, subcutaneous tumors were excised and further analyzed. To establish the ferroptosis-related subcutaneously implanted tumor model, 1 × 10^7^ cells were harvested and re-suspended in 150 μl of PBS and injected subcutaneously into 4–6-week-old male BALB/c nude mice. Tumor volume was measured as (tumor width^2^ × tumor length)/2 and sorafenib (30 mg/kg/day) intra-gastric administration began on day 10. Finally, subcutaneous tumors in nude mice were excised on day 35 and further analyzed. All animal studies were approved by the Institutional Animal Care and Use Committee of Nanjing Drum Tower Hospital and Nanjing University, in accordance with the guidelines for the care and use of laboratory animals.

### Western blotting

RIPA Lysis Buffer (Beyotime Biotechnology, Shanghai, China) was used to lyse cells and liver tissue. Protein concentrations were quantified using a BCA kit (Beyotime Biotechnology, Shanghai, China). Proteins were separated by sodium dodecyl sulfate (SDS)-PAGE on 10% gels and transferred to poly-vinylidene difluoride (PVDF) membranes (Sigma-Alcdrih, St. Louis, MO, USA). After overnight incubation with various primary antibodies, including anti-HMGCL (1:1000, 16898-1-AP, Proteintech), GAPDH (1:2000, 10494-1-AP, Proteintech), DPP4 (1:1000, YT5707, Immunoway), H3 (1: 1000, 17168-1-AP, Proteintech), H3K9ac (1: 1000, ab32129, Abcam), H4 (1: 1000, 16047-1-AP, Proteintech), H4ac (1: 1000, 39026, Activemotif), Pan anti-acetyllysine (1: 1000, PTM-105, Jingjie PTM BioLab), Anti-LC3 (1: 1000, 4599, Cell Signaling Technology), Anti-P62 (1: 1000, 16177, Cell Signaling Technology), P16 (1: 1000, ab189034, Abcam), P21 (1: 1000, ab107099, Abcam), NOX1 (1: 1000, 17772-1-AP, Proteintech), EGFR (1: 1000, 66455-1-Ig, Proteintech). Then incubated for 2 h in the presence of secondary antibody (1: 2000, A0208, Beyotime) and washed 3 times with TBST for 5 min. The signals were detected using the ECL chemiluminescence system and analyzed by ImageJ Lab software.

### Cell culture

The human HCC cell lines (Huh7, Hep3B, HepG2, MHCC-LM3, MHCC-97L, and MHCC-97H) were purchased from Shanghai Cell Bank of Chinese Academy of Science (Shanghai, China). They were cultured in DMEM medium (Gibco, Grand Island, NY, USA) supplemented with 10% fetal bovine serum (FBS) and grown at 37 °C in a 5% CO_2_ environment. The content of anagliptin used in the study was 20 µM.

### Statistical analysis

Data were analyzed with Student's t test or ANOVA. Unless otherwise stated, all data are presented as mean ± SEM. For all tests, *p* < 0.05 was considered statistically significant (*). Statistical analyses were performed in GraphPad Prism 8.

### Additional methods

For further details regarding the materials and methods used, please refer to the supplementary information.

## Results

### Proteome data and clinical validation link HMGCL to HCC suppression

To investigate the suppressive proteins in the HCC process, we analyzed the differential proteome profile between HCC tissues and paired adjacent tissues from 101 early-stage HCC patients published previously [26], HMGCL was identified to be enriched in 6 of top 10 biological processes in down-regulated protein dataset by gene ontology (GO) analysis (Supplementary Fig. S1A). Meanwhile, HMGCL was down-regulated in HCC tumor lesions according to the results of proteomics data in public databases (n = 101) (Supplementary Fig. S1B, C). According to this proteomic dataset, HMGCL was averaged approximately ~ 1.7-fold downregulated in HCC tumors, and samples with two fold down-regulation accounted for approximately 50.5% (51/101) in early-stage HCC patients (Supplementary Fig. S1B, C). Genomic enrichment analysis (GSEA) of this proteomic data showed that the low expression of HMGCL was associated with oncogenic EMT and E2F signaling (Supplementary Fig. S1D, E), whereas the enriched dataset with high HMGCL was associated with bile acid metabolism and peroxisome, which are involved in anti-tumor activity (Supplementary Fig. S1F, G) [27, 28].

**Fig. 1:**
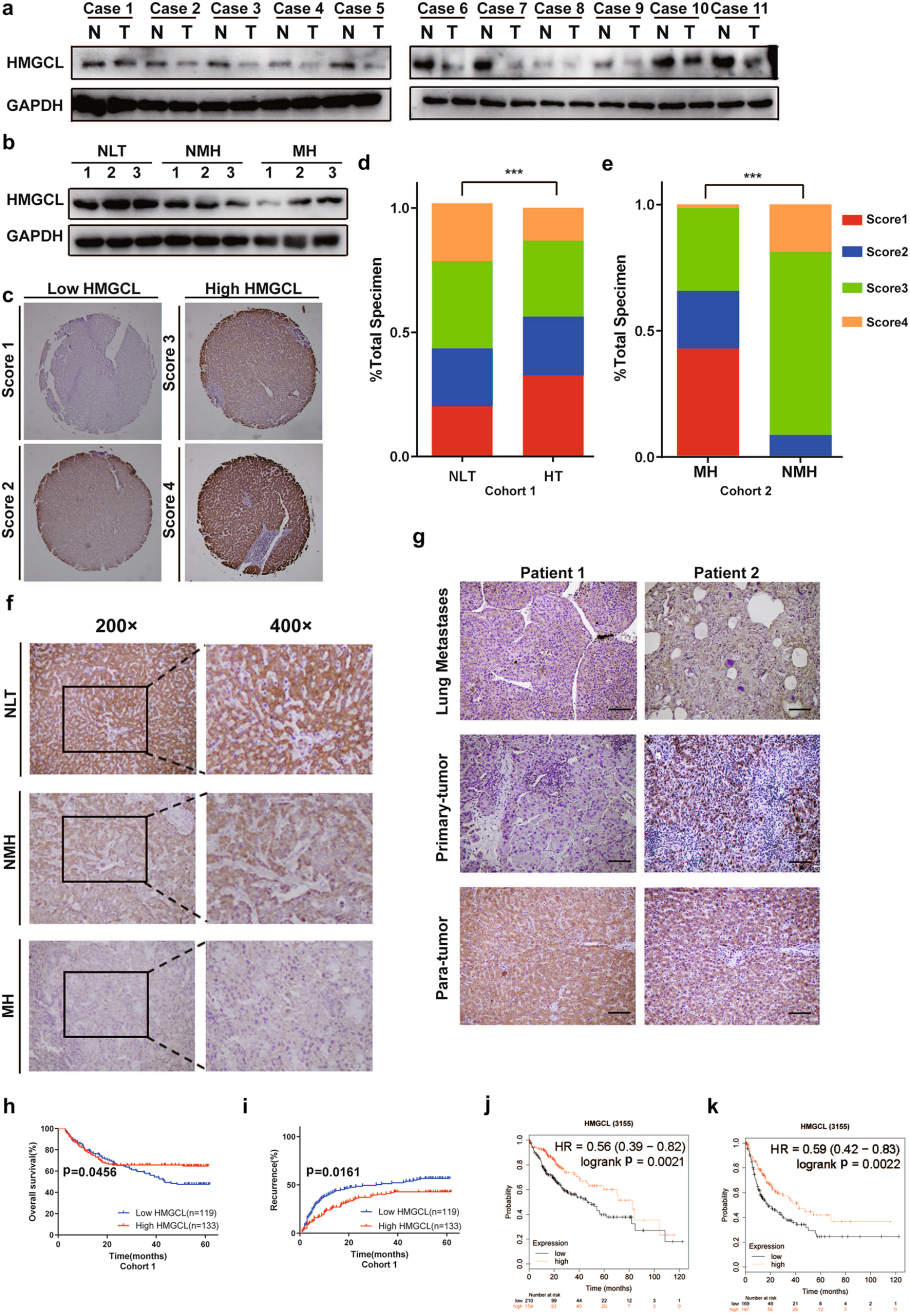
Down-regulation of HMGCL is correlated with HCC metastasis and proliferation. **A** Western blot of HMGCL protein levels in 11 HCC tissue (T) and paired normal liver tissues (N). **B** Western blot of HMGCL protein levels in paired normal liver tissues (NLT, *n* = 3), non-metastatic HCC tissues (NMH, n = 3) and metastatic HCC tissues (MH, *n* = 3). **C** Scores indicate HMGCL protein levels in representative tumor tissues tested by immuno-histochemical (IHC) staining. The score was counted by intensity and percentage of staining cells. **D**, **E** Quantification of HMGCL expression level based on the IHC scores of cohort 1 (including 253 HCC tissue (HT) and paired NLT) and cohort 2 (including 40 MH and 40 NMH). **F** Representative images of IHC staining of HMGCL from three NLT, NMH, or MH specimens. **G** Representative images of IHC staining of HMGCL from 2 cases of the primary tumor, lung metastases and para-tumor. **H** The OS of HCC patients (cohort 1) with different expression levels of HMGCL. **I** The RR of HCC patients (cohort 1) with different expression levels of HMGCL. **J** The OS of HCC patients with different expression levels of HMGCL assessed via Kaplan–Meier (K–M) analysis. **K** The PFS of HCC patients with different expression levels of HMGCL assessed via K–M analysis. Each experiment was performed at least three times, all data were showed as mean ± SD. *p < 0.05, **p < 0.01, ***p < 0.001, ns as no significance

The clinical relevance of HMGCL expression was analyzed in HCC clinical samples by IHC and WB. We quantified the HMGCL expression level of HCC in 11 pairs of HCC and matched non-tumor liver tissues (NLTs) and other samples including 3 cases of normal liver tissues, 3 cases of non-metastatic HCCs and 3 cases of metastatic HCCs by WB (Fig. 1A, B). We found that HMGCL expression levels were significantly lower in HCC lesions than in NLTs (Fig. 1A, B). Then, we evaluated HMGCL expression according to the intensity and breadth of staining areas by IHC in two independent cohorts of HCC samples, score 1 and score 2 were identified as HMGCL^Low^ and score 3 and score 4 as HMGCL^High^. We found that scoring of HMGCL was significantly lower in HCC tissues (HTs) compared to adjacent NLTs in cohort 1 (Fig. 1C, D). Furthermore, the scorings in metastatic HCC tissues (MHs) were significantly lower than in non-metastatic HCC tissues (NMHs) in cohort 2 (Fig. 1E, F). Further analysis of the HMGCL expression in the 2 cases of lung metastatic HCC indicated that HMGCL protein levels were also significantly decreased in lung metastases lesion (Fig. 1G), suggesting that HMGCL depletion was correlated with HCC aggressiveness. Through the analysis based on TCGA database, HMGCL expression was heterogeneous in pan-cancer (Supplementary Fig. S2A, B), leading to multiple HMGCL effects in different tumor types.

### HMGCL expression is associated with outcomes of HCC patients

To further validate the association between HMGCL expression and HCC patients’ prognosis, we performed IHC utilizing a tissue microarray (TMA) from 252 HCC tissues from cohort 1 (Fig. 1C, D) with clinicopathological information that listed in Table 1. HMGCL^Low^ patients were associated with microvascular invasion, elevated APF levels, increased tumor size, poorly differentiated tumors and pTNM characteristics, decreased overall survival (OS) and increased recurrence rates (RR) (Table 1 and Fig. 1H, I). Reduced OS and poor progression-free survival (PFS) were further confirmed in another independent cohort of HCC patients from The Cancer Genome Atlas (TCGA) (Fig. 1J, K). Taken together, these results show that downregulation of HMGCL was significantly associated with poor prognosis in HCC patients.

### HMGCL suppresses HCC metastasis and proliferation

**Fig. 2:**
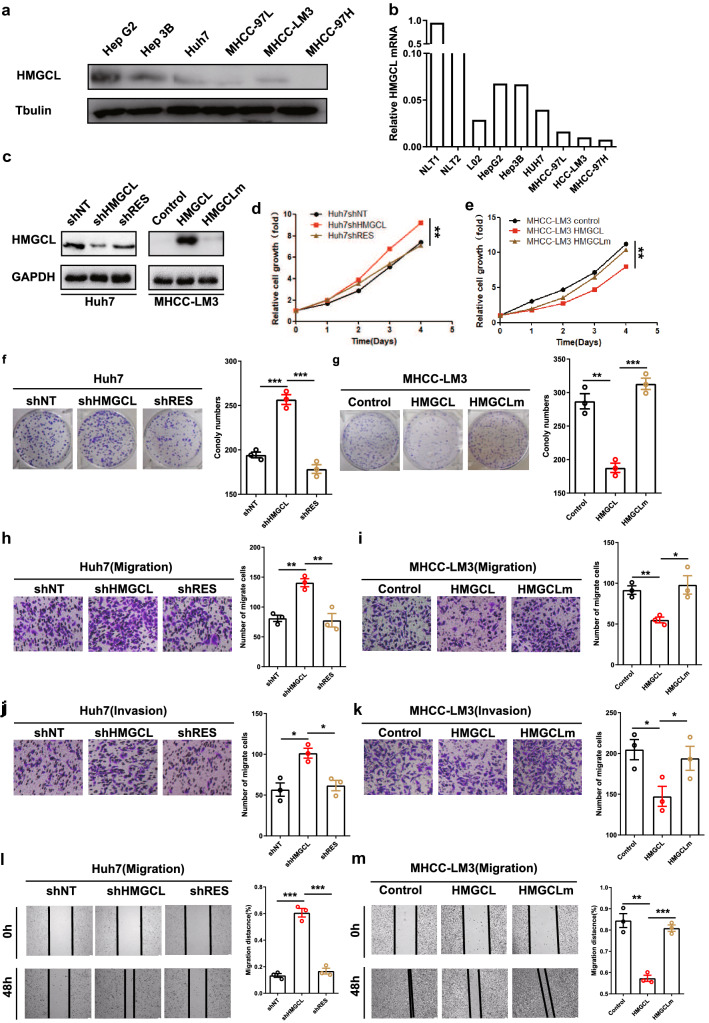
HMGCL suppresses HCC metastasis and proliferation in vitro. **A** Western blot of HMGCL protein in HCC cell lines with different metastatic and proliferation potentials. **B** qRT-PCR of HMGCL mRNA in HCC cell lines with different metastatic and proliferation potentials. **C** Confirmation of HMGCL knockdown (KD, shHMGCL), re-expression (shRES), overexpression (HMGCL) and overexpression with mutation site (HMGCLm) in HCC cell lines. **D**, **E** The effect of HMGCL gain- or loss-of-function on in vitro proliferation utilized CCK8 assay. The relative cell number was counted as a fold change of Day 0. **F**, **G** The effect of HMGCL gain-or-loss-of-function on in vitro proliferation utilized colony-formation assay. Cell numbers were counted at day 14. **H**–**M** The effect of HMGCL gain-or-loss-of-function on in vitro migrated and invaded cell numbers was measured utilizing trans-well and wound-healing assay. Each experiment was performed at least three times, all data were shown as mean ± SD. *p < 0.05, **p < 0.01, ***p < 0.001, ns as no significance

Next, we performed gain-and loss-of function studies to assess the functional role of HMGCL on HCC process both in vitro and in vivo. WB result showed that HMGCL expression was higher in low metastatic HCC cell lines, such as HepG2, Hep3B and Huh7, than in high metastatic HCC cell lines, such as MHCC-LM3, MHCC-97H and MHCC-97L (Fig. 2A, B). Then, we assessed the role of HMGCL on the proliferation, migration and invasion in vitro. We overexpressed HMGCL in MHCC-97H and MHCC-LM3 cells, whereas HMGCL-specific short hairpin (sh) RNAs were used to knockdown HMGCL in Huh7 and Hep3B (Fig. 2C and Supplementary Fig. S3A). HMGCL overexpression (HMGCL^OE^) resulted in impaired proliferation of MHCC-97H and MHCC-LM3 cells, in contrast, HMGCL knocked down (HMGCL^KD^) induced Hep3B and Huh7 proliferation (Fig. 2D–G and Supplementary Fig. S3B-E). Meanwhile, HMGCL downregulation significantly enhanced the migration and invasion capacity of HCC cells (Fig. 2H, J, L and Supplementary Fig. S3F, H). In contrast, HMGCL^OE^ MHCC-97H and MHCC-LM3 decreased cell migration and invasion (Fig. 2I, K, M and reversed these trends in Supplementary Fig. S3G, I). To examine whether HMGCL re-expression reversed proliferation, migration, and invasion in HCC cells and excluded the off-target effects, we reintroduced engineered cDNA (shRES) for shRNA-insensitive HMGCL into HMGCL-silenced cells (Fig. 2C and Supplementary Fig. S3A), we found that re-expression of HMGCL reversed the enhancement of HCC cell migration and invasion induced by HMGCL silencing (Fig. 2D, F, H, J, L and Supplementary Fig. S3B, D, F, H). We further mutated the enzymatic activity sites of HMGCL with D42A and H233A (HMGCLm), and found that HMGCLm rescued the reduction of proliferation, migration and invasion capacity of the cells mediated by HMGCL^OE^ (Fig. 2E, G, I, K, M and Supplementary Fig. S3C, E, G, I). In conclusion, these results above support the idea that depletion of HMGCL in HCC cells leads to the promotion of proliferation, migration and invasion of these cells in vitro.

**Fig. 3:**
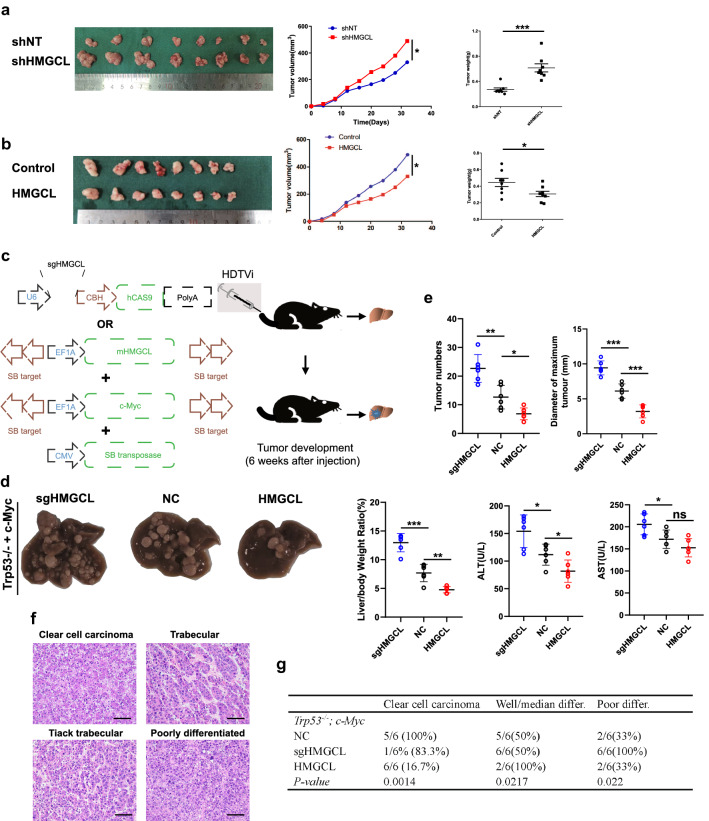
HMGCL suppresses HCC metastasis and proliferation in vivo. **A**, **B** The effect of HMGCL gain-or-loss-of-function of tumor volume and weight in subcutaneous xenograft models was shown. **C** Trp53^loxp/loxp^ mice were utilized to establish a spontaneous HCC model by tail vein injection c-Myc and NC/HMGCL/sgHMGCL plasmids. **D** Spontaneous HCC samples of Trp53^Δhep/Δhep^ mice with different plasmids. **E** The number of tumors, the maximum tumor diameter, liver weight/body ratio, ALT, AST in Trp53^Δhep/Δhep^ mice with different HMGCL expression. **F**, **G** Typical pathological types and proportions of spontaneous HCC models. Each experiment was performed at least three times, all data were shown as mean ± SD. *p < 0.05, **p < 0.01, ***p < 0.001, ns as no significance

To confirm the in vitro results, we further investigated the effect of altered HMGCL expression on tumor proliferation and metastasis in vivo. In a subcutaneous xenograft model, HMGCL^OE^ inhibited tumor growth while HMGCL^KD^ induced this process (Fig. 3A, B). In tail vein injection lung metastasis model, the number of lung metastases was significantly increased in mice treated with shHMGCL cells (Supplementary Fig. S4A) and decreased in mice injected with HMGCL overexpressing cells (Supplementary Fig. S4B). Ki67 and PCNA IHC also confirmed the enhanced proliferation after HMGCL^KD^ and reduced in HMGCL^OE^ (Supplementary Fig. S4C).

### HMGCL inhibits *Trp53*^*Δhep/Δhep*^*; c-Myc*-driven liver tumorigenesis

To further examine HMGCL functions in the cancerization course from normal hepatocytes to cancer cells in vivo, we generated hepatocyte-specific deletion of *Trp*53 mice (*Trp*53^*Δhep/Δhep*^) by crossing *Trp53*^*flox/flox*^ with *Alb-Cre* mice, which mimicked 58% of HCC patients possessing the *Trp*53 mutation as previously described previously [29]. Then we cloned a CRISPR-Cas9 fraction of HMGCL (sgHMGCL-Cas9) and the HMGCL cDNA into the Sleeping Beauty (SB) vector (NC) and hydro-dynamically injected different combinations of pSB-U6-gHMGCL-Cas9, pSB-EF1α-HMGCL, pSB-EF1α-Myc and CMV-SB10 transposase plasmids via the tail vein in *Trp53*^*Δhep/Δhep*^ mice (Fig. 3C). All the tumor sections were positively stained by hepatocyte lineage marker HNF4α, and negatively stained by cholangiocyte lineage marker KRT19 (Supplementary Fig. S4D), demonstrating that *Trp*53^*Δhep/Δhep*^ and *c-Myc*-driven tumors were HCC instead of intrahepatic cholangiocarcinomas. At 6 weeks after injection, HMGCL depletion led to increased tumor number, size and liver weight/body ratio, as well as aspartate aminotransferase (AST) and alanine transaminase (ALT) concentration in serum (Fig. 3D, E). HCC in *Trp53*^*Δhep/Δhep*^; *c-Myc*-driven mice exhibited a range of histological grades, from well to poor differentiated HCCs (Fig. 3F), expression progressively decreased from benign hepatic adenomas to poorly differentiated HCCs (Fig. 3G), similar to the patient’s information. These results suggested that HMGCL depletion led to liver damage and tumorigenesis.

### HMGCL enhances HCC cell ferroptosis vulnerability

**Fig. 4:**
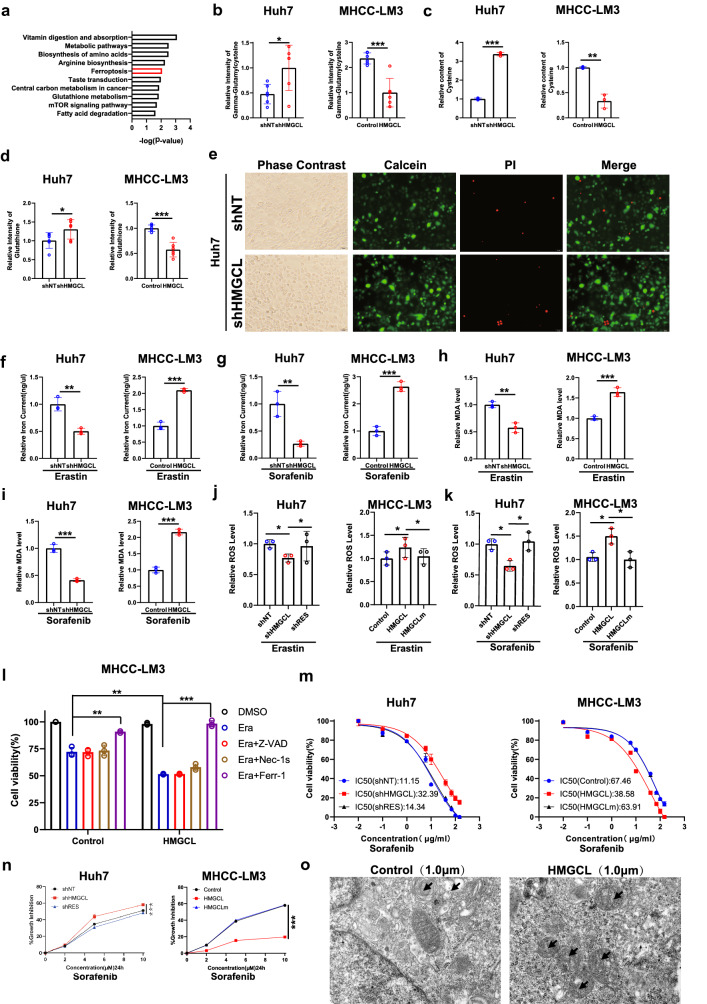
HMGCL regulates ferroptosis-related metabolites in HCC cells. **A** Metabolomics results show that altered expression of HMGCL could be involved in ferroptosis-related metabolic processes. **B**–**D** Altered expression of HMGCL is involved in the regulation of γ-glutamyl-cysteine, cysteine and glutathione levels. **E** Knocking down HMGCL did not change the proportion of PI (red) positive cell. **F**, **G** Altering the expression of HMGCL can affect the iron current in HCC cells. **H**, **I** Altering the expression of HMGCL can affect the MDA in HCC cells. **J**, **K** Altered expression of HMGCL is involved in the regulation of ROS levels. **L** Over-expression of HMGCL increased the sensitivity of HCC cells to ferroptosis. **M** Altering the expression of HMGCL can affect the sensitivity of HCC cells to sorafenib. **N** Altering the expression of HMGCL can affect the sensitivity of HCC cells to sorafenib. **O** Effect of altered HMGCL expression on mitochondrial morphology in MHCC-LM3 cells. Each experiment was performed at least three times, all data were shown as mean ± SD. *p < 0.05, **p < 0.01, ***p < 0.001, ns as no significance

To investigate the mechanism of HMGCL-induced HCC suppression, we examined metabolome in shHMGCL, shNT, HMGCL and control cell lines. Then, we performed enrichment utilizing metabolites that were simultaneously altered in the HMGCL^KD^ and HMGCL^OE^ groups. In the top 10 enriched pathways, we focus on ferroptosis—a form of RCD (Fig. 4A). Previous studies have shown the increased of ketone was considered to activate autophagy, another kind of RCD, of the tumor [12, 19]. We want to further explore whether HMGCL could affect HCC ferroptosis and whether this process was autophagy-dependent. Therefore, we examined ferroptosis-related metabolites, and the results showed that γ-glutamyl-cysteine, cysteine and glutathione were upregulated in the HMGCL^KD^ group and downregulated in the HMGCL^OE^ group (Fig. 4B–D). However, knocking down the expression of HMGCL alone could not affect the number of cell death (Fig. 4E). Therefore, we hypothesized that HMGCL could influence the sensitivity of HCC cells to ferroptosis. Furthermore, the level of iron current, malondialdehyde (MDA) and reactive oxygen species (ROS) were decreased in HMGCL^KD^ cells under the pressure of sorafenib and erastin treatment, which are referred as ferroptosis inducers (Fig. 4F–K). To further determine the active role of HMGCL in ferroptosis, we evaluated erastin-induced cell death. Erastin prompted about 50% cell death in HMGCL^high^ group, and about 30% cell death in control group. A ferroptosis inhibitor ferrostatin-1, but not an apoptosis inhibitor Z-VAD-FMK (Z-VAD) or necroptosis inhibitor necrostatin-1 s (Nec-1 s), had the ability to rescue the MHCC-LM3 cell viability which impaired by erastin (Fig. 4L), and similar results were observed in Huh7 cells (Supplementary Fig. S5A). The result indicated that HMGCL mediated ferroptosis rather than other commonly RCD. In addition, HMGCL knockdown decreased vulnerabilities of sorafenib, whereas HMGCL overexpression reversed this trend (Fig. 4M, N). Then, the mitochondrial structure was atrophied after erastin treatment in HMGCL^OE^ cells was observed utilized transmission electron microscopy (TEM) (Fig. 4O). To determine whether HMGCL mediated autophagy-dependent ferroptosis, the expressions of LC3 and p62 were tested via WB, but there is no significant change with HMGCL alteration (Supplementary Fig. S5A). In summary, our results showed that HMGCL increased vulnerabilities of sorafenib and induced autophagy-independent ferroptosis.

### HMGCL regulates the expression of DPP4 through β-OHB-dependent acetylation

**Fig. 5:**
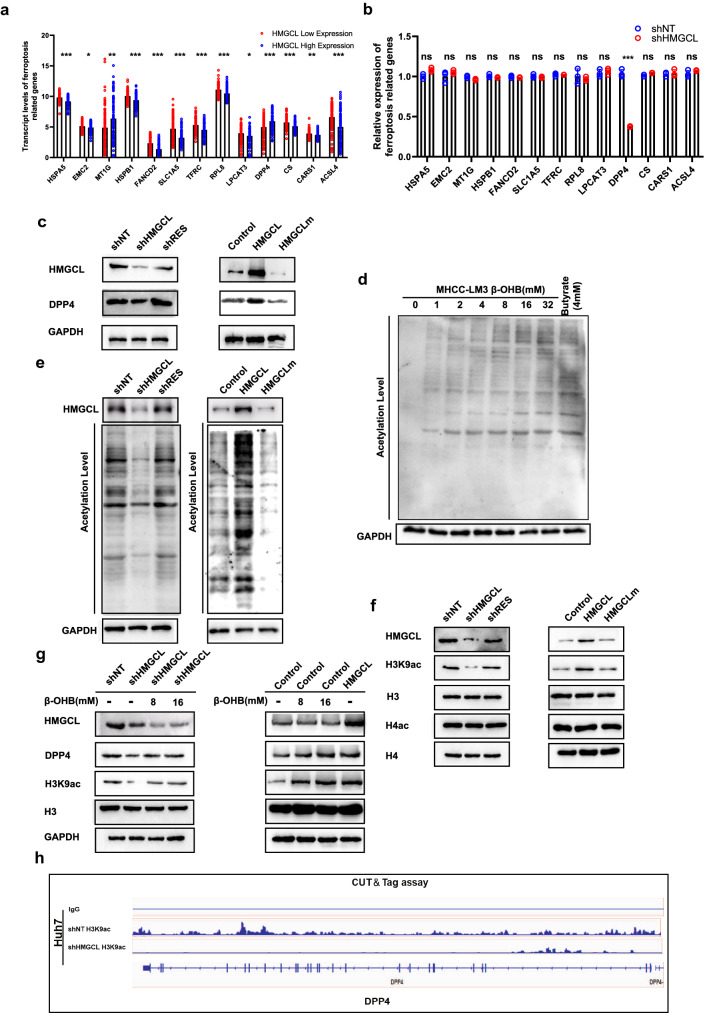
HMGCL regulates DPP4 by mediating acetylation. **A** Based on the information of patients with high expression of HMGCL (*n* = 93) and patients with low expression of HMGCL (*n* = 93), ferroptosis-related genes which significantly related to HMGCL were predicted by bioinformatics. **B**, **C** Altered HMGCL expression could affect the transcription and expression of DPP4 detected by qRT-PCR and western blot. **D** Effect of increasing β-OHB on the total acetylation level of MHCC-LM3 (butyrate as a positive control). **E** Altering the expression of HMGCL can change the total acetylation level of HCC cells. **F** Effect of altered HMGCL expression on H3K9ac in HCC cells. **G** Increasing exogenous β-OHB can affect the expression of DPP4. (H) CUT & Tag method to detect the effect of altered HMGCL expression on the DPP promoter

To investigate the mechanisms that HMGCL regulates ferroptosis vulnerability, we used bioinformatics to predict HMGCL-related ferroptosis genes. Distinctly different ferroptosis genes were sought between HMGCL^high^ HCC tissues (*n* = 93) and HMGCL^low^ expression HCC tissues (*n* = 93) (Fig. 5A). The correlation between HMGCL and ferroptosis related genes via qPCR and WB. The results supposing that DPP4, a glutathione suppressant [30–33], was positively correlated with HMGCL expression (Fig. 5B, C). As reported previously, HMGCL is responsible for converting HMG-CoA into β-OHB and acetoacetate (AcAc) [16–18] (Supplementary Fig. S6A). Moreover, exogenous ketone bodies could change the acetylation level of protein [34]. First, we examined modulating HMGCL expression on cellular AcAc and β-OHB level. When HMGCL was silenced, the cellular AcAc and β-OHB levels decreased, whereas cellular AcAc and β-OHB levels increased after HMGCL overexpression (Supplementary Fig. S6B, C). WB results showed that cellular acetylation level was increased with β-OHB elevating (Fig. 5D). WB results further confirmed that HMGCL also positively regulated cellular acetylation levels (Fig. 5E and Supplementary Fig. S7A). Previous researches thought β-OHB could promote the acetylation of histone H3K9 [35], a site which reduces DPP4 expression by methylation [32]. Hence, we explored whether H3K9ac also affected DPP4 expression. WB showed that HMGCL increased H3K9ac level and had no effect on total H4ac level, suggesting that HMGCL precisely modulated acetylation on histone H3K9 (Fig. 5F). β-OHB incubation showed that HMGCL depletion reduced H3K9ac and DPP4, and HMGCL overexpression induced these two proteins with dose-dependent β-OHB level (Fig. 5G and Supplementary Fig. S7B). Next, we further performed CUT & Tag assay upon HMGCL depletion and found that HMGCL depletion attenuated the acetylation of H3K9, which impaired DPP4 transcription (Fig. 5H).

DPP4 is heterogeneous in ferroptosis of colon cancer, which is dependent on the cellular localization of DPP4 and the genotype of *Trp*53 [30, 33]. Our result showed that HMGCL altered DPP4 expression was not dependent on subcellular localization (Supplementary Fig. S8A, B). Meanwhile, the cell lines used in this study were *Trp*53-mutated Huh7 (c.659A > G) and MHCC-LM3 (c.151G > T), and HMGCL-modulated DPP4 expression is not *Trp*53 mutant-dependent (Supplementary Fig. S8A, B). Previous research thought β-OHB could regulate the process of cellular senescence through acetylation [35], but we found that HMGCL-mediated acetylation did not affect cellular senescence in HCC (Supplementary Fig. S5C, D). Taken together, HMGCL increased the acetylation and induced ferroptosis in HCC cells.

### DPP4 reduction reversed HMGCL-induced ferroptosis vulnerability

**Fig. 6:**
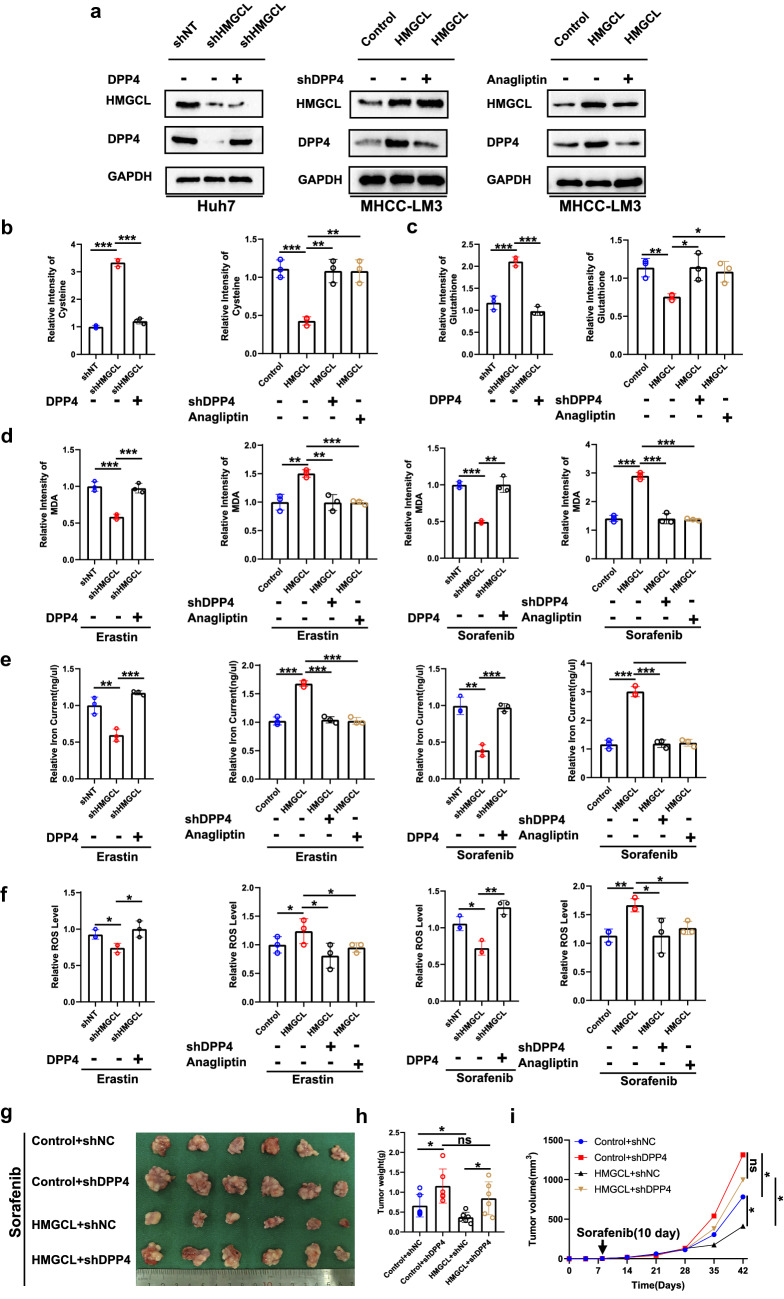
Altering DPP4 expression can affect the HMGCL-mediated ferroptosis sensibility. **A** The expression of shHMGCL cells transfected with DPP4, HMGCL cells transfected with shDPP4, and HMGCL cells treated with anagliptin. **B**, **C** Effect of overexpression of DPP4 on cysteine and glutathione levels in shHMGCL cells. **D**–**F** Effect of transfection with DPP4 and treated with anagliptin on MDA, iron current and ROS levels in HMGCL cells. **G**–**I** The data, including tumor volume and weight, of the sorafenib-induced ferroptosis model which were influenced by altered HMGCL expression. Each experiment was performed at least three times, all data were showed as mean ± SD. *p < 0.05, **p < 0.01, ***p < 0.001, ns as no significant

To investigate whether DPP4 rescued the effect of HMGCL on ferroptosis, we re-expressed DPP4 in HMGCL^KD^ cells or re-depleted DPP4 in HMGCL^OE^ cells (anagliptin was selected as a positive control of DPP4 deletion) (Fig. 6A) [36]. When DPP4 was re-expressed into the HMGCL^KD^ cells, it significantly suppressed cysteine, glutathione, and elevated MDA, iron current and ROS level, whereas DPP4 deprivation in HMGCL^OE^ cell reversed these trends, compared with non-anagliptin treated groups (Fig. 6B–F). Then, we found that DPP4 knockdown in the control and HMGCL^OE^ cells significantly reduced the ferroptosis process commitment with sorafenib vulnerabilities in vivo (Fig. 6G–I). Furthermore, DPP4 knockdown partially restored the proliferation and metastasis of HMGCL^OE^ cells. Similarly, DPP4 re-expression inhibited the proliferation and metastasis of HMGCL^KD^ cells (Supplementary Fig. S9A-H).

As described previously, DPP4 increased ferroptosis vulnerability through interaction with NADPH oxidase 1 (NOX1) [33], we investigated the relationship between DPP4 and NOX1 in HCC cells. The results showed that NOX activity was significantly reduced following HMGCL depletion, and NOX activity was significantly stimulated with HMGCL overexpression, suggesting that HMGCL-induced DPP4 might activate NOX1 to induce ferroptosis in HCC cells (Supplementary Fig. S8C, D). Subsequently, we demonstrated that DPP4 bind to NOX1, might be promoted by HMGCL (Supplementary Fig. S8E).

At last, we tested the expression of 4-HNE in different group, the results showed that the DPP4 depletion could impair the ferroptosis sensibility enhanced by HMGCL up-regulation (Supplementary Fig. S8F). The similar results could be observed in the Trp53^Δhep/Δhep^; c-Myc-driven mice, although we did not utilize the erastin, and sorafenib induced the ferroptosis (Supplementary Fig. S8G).

### Up-regulation of HMGCL is associated with elevated expression of total acetylation, H3K9ac and DPP4 in HCC tissues

**Fig. 7:**
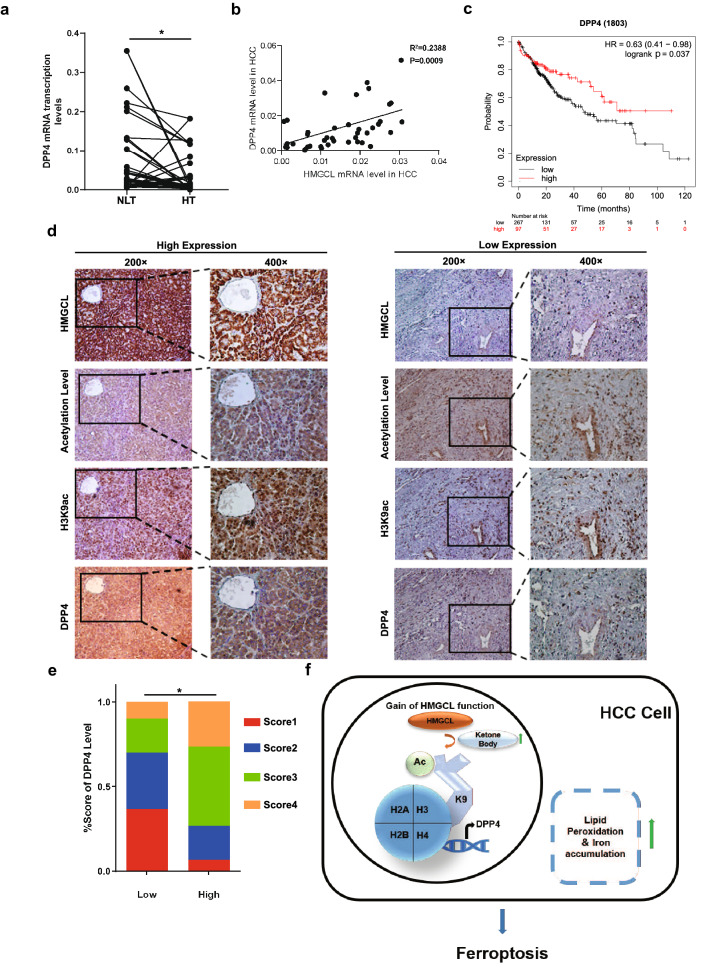
Up-regulation of HMGCL is correlated with increased histone acetylation and DPP4 expression in HCC tissues. **A** The transcription levels of DPP4 in 40 paired samples of HCC tissues (HT) and matched normal liver tissues (NLT) (B) Correlation of transcription levels of DPP4 with transcription levels of HMGCL in 40 pairs of HCC samples. **C** The OS of HCC patients with different expression levels of DPP4 assessed via Kaplan–Meier analysis (*n* = 364). **D**, **E** The correlation of DPP4 expression and HMGCL expression in 50 cases of (including 25 low HMGCL expression specimens and 25 high HMGCL expression specimens) HCC samples. **F** Schematic diagram depicting the regulation of HMGCL in HCC cells

Finally, we validated the relationship by qRT-PCR and IHC assay. Consistent with the WB results, total acetylation and H3K9ac expression levels were positively correlated with the HMGCL expression levels (Supplementary Fig. S10A-D). qRT-PCR result showed that DPP4 were down-regulated in HT compared with NLT (Fig. 7A), and DPP4 expression was positively correlated with HMGCL (Fig. 7B). The correlation between DPP4 transcription levels and clinical OS of patients was mapped based on TCGA dataset (*n* = 364) and Kaplan–Meier plotter showed that low DPP4 expression was associated with poor OS, which was similar with the results of HMGCL (Fig. 7C). Then the correlation between HMGCL and the level of cellular acetylation, H3K9Ac and DPP4 was investigated using IHC assay. As expected, HMGCL^High^ was associated with up-regulation of total histone acetylation, H3K9Ac and DPP4, whereas HMGCL^Low^ was associated with down-regulation of these markers (Fig. 7D–E).

Taken together, our findings outline the HMGCL-DPP4 axis in HCC progression. HMGCL increases acetylation level on histone H3K9 site, leading to interaction with DPP4 promoter and stimulation of DPP4 transcription (Fig. 7F). DPP4 induces ferroptosis and further inhibits HCC proliferation and metastasis.

## Discussion

The β-OHB is closely related to various cellular processes in cell growth and mitosis. HMGCL as the key enzyme to promote the conversion of HMG-CoA to ketone bodies, had been linked to several cancers [17, 18]. However, the physiological role of HMGCL has not been well determined. In the current study, we found that the transcription and expression of HMGCL were significantly down-regulated in HCCs. Furthermore, we detected correlation between HMGCL depletion and poor prognosis of HCC patients, including microvascular invasion, elevated APF level, increased tumor size, poor differentiated stage and poor pTNM characteristic. Functionally, our study demonstrated overexpression of HMGCL inhibited the proliferation and metastasis of HCC. In *Trp53*^*flox/flox*^*; c-Myc* driven HCC mice model, HMGCL deprivation accelerates tumor growth. HMGCL has heterogeneous effects on multiple cancer types [16–18]. Consistently, pan-cancer gene expression analysis showed that HMGCL mRNA was differentially expressed in different tumor types (Fig S2A, B). While some tumor types, such as bladder urothelial carcinoma and uterine corpus endometrial carcinoma, showed elevated HMGCL expression, other tumor types, such as pheochromocytoma and para-ganglioma as well as cholangial carcinoma, showed decreased HMGCL expression (Fig S2A, B). Our study showed that HMGCL expression was significantly down-regulated in HCC. However, HMGCL protein was detected in the majority of NLT. Therefore, HMGCL might be a potential suppressor for anti-HCC activity.

Mechanistically, we identified DPP4 as a transcriptional target of HMGCL, which is located at the plasma membrane, where it functions as a serine exopeptidase cleaves X-proline dipeptides from the N-terminus. In addition to its role in diabetes, the biological role of DPP4 in various types of cancers, including HCC and ccRCC, has also been investigated [32, 37]. DPP4 was thought to be a key enzyme in the process of ferroptosis, as it elevated lipid ROS, limited glutathione level and stimulated ferroptosis [29–32]. And, we found that DPP4 not only downregulated glutathione but also cysteine level. Additionally, DPP4 binds to NOX1 to form a DPP4–NOX1 complex, which regulates the NOX activities and ferroptosis. This result showed the potentiality of HMGCL in ferroptosis vulnerability via regulating DPP4 expression. In fact, DPP4 inhibitors are considered to inhibit tumor development in some tumors. This may be caused by tumor heterogeneity, gene mutation backgrounds, metabolism backgrounds and drug stimulation backgrounds.

As ketone bodies increase histone acetylation and DPP4 transcription could be enhanced through histone acetylation [38], we tested whether HMGCL induces DPP4 expression via histone acetylation. In this study, exogenous ketone bodies elevated the total acetylation level in HCC cell lines. H3K9ac is an acetylated histone site could be modified by ketone bodies. Epigenetic modifications at the H3K9 are thought to be involved in the transcriptional activity of DPP4 [32]. We have confirmed that HMGCL regulated DPP4 expression through H3K9ac. These results provide a novel mechanism for regulating the DPP4 expression, which is distinct from the traditional mechanism, such as HMGCS2 inhibits tumor growth and metastasis by ketone-dependent autophagy [12]. Our study also demonstrated that HMGCL, another key enzyme in ketone body synthesis, could enhance ferroptosis vulnerability, a term of cell programmed death in HCC cells with a non-autophagic-dependent pathway.

## Conclusion

In conclusion, our study shows that the downregulation of HMGCL is an important feature of HCC proliferation and metastasis. Mechanistically, HMGCL increases β-OHB level and induces H3K9 acetylation, promoting the transcription of DPP4. Thus, the HMGCL-DPP4 axis induced ferroptosis and impaired the resistance of sorafenib and erastin to treat HCC. In addition, upregulation of HMGCL and DPP4 has been linked to a better prognosis in human HCC cohorts, and our study might provide mechanistic insights into these clinical applications.

## Supplementary Information

Below is the link to the electronic supplementary material.

Supplementary file1 (DOCX 11075 KB)

## Data Availability

All relevant data from this research are available by contacting the corresponding author.

## Abbreviations

AFP: α-Fetoprotein
ANOVA: Analysis of variance
HMGCL: Hydroxymethylglutaryl-CoA lyase
WB: Western blot
EMT: Epithelial–mesenchymal transitions
GSH: Glutathione
HCC: Hepatocellular carcinoma
HNF4α: Hepatocyte nuclear factor 4α
KO: Knockout
OE: Over-expression
Trp53: Transformation-related protein 53
qRT-PCR: Quantitative reverse-transcription PCR
SB: Sleeping beauty
DPP4: Dipeptidyl peptidase 4
IHC: Immunohistochemistry
