# Correction to: HMGCL‑induced β‑hydroxybutyrate production attenuates hepatocellular carcinoma via DPP4‑mediated ferroptosis susceptibility

**DOI:** 10.1007/s12072-022-10476-8

**Published:** 2023-01-13

**Authors:** Xiaohan Cui, Xiao Yun, Meiling Sun, Renzhi Li, Xiajie Lyu, Yuanxiang Lao, Xihu Qin, Wenbin Yu

**Affiliations:** 1 Department of Gastrointestinal Surgery, General Surgery, Qilu Hospital, Cheeloo College of Medicine, Shandong University, Jinan, 250012 Shandong People’s Republic of China; 2 Nanjing Medical University, Nanjing, 211166 Jiangsu People’s Republic of China; 3 Department of General Surgery, The Affiliated Changzhou No. 2 People’s Hospital of Nanjing Medical University, 68 Pohu Middle Road, Changzhou, 213000 Jiangsu People’s Republic of China; 4 Department of Hepatobiliary Surgery, The Affiliated Drum Tower Hospital of Nanjing University Medical School, 321 Zhongshan Road, Nanjing, 210008 Jiangsu People’s Republic of China; 5 Gangarosa Department of Environmental Health, Rollins School of Public Health, Emory University, Atlanta, GA 30332 USA

Correction to: Hepatology International 10.1007/s12072-022-10459-9

In this article, the order that the authors appeared in the author list was incorrect.

The order of authors should be:

Xiaohan Cui1,2 · Xiao Yun3 · Meiling Sun4 · Renzhi Li3 · Xiajie Lyu5 · Yuanxiang Lao4 · Xihu Qin2,3. Wenbin Yu1.

The original article has been updated accordingly.

