# Supplementary material for: HMGCL-induced β-hydroxybutyrate production attenuates hepatocellular carcinoma via DPP4-mediated ferroptosis susceptibility
Source: Hepatol Int. 2022 Dec 12;17(2):377–92. doi: 10.1007/s12072-022-10459-9 (PMC10119270; doi:10.1007/s12072-022-10459-9)
Supplement: Supplementary file 1 — Supplementary file1 (DOCX 11075 KB) [file 12072_2022_10459_MOESM1_ESM.docx]

**Supporting information for**

**HMGCL-induced β-Hydroxybutyrate Production Attenuates Hepatocellular Carcinoma via DPP4-mediated Ferroptosis** **Susceptibility**

Xiaohan Cui^1,2^, Xiao Yun^3^, Meiling Sun^4^, Renzhi Li^3^, Xiajie Lyu^5^, Yuanxiang Lao^3*^, Wenbin Yu^1*^, Xihu Qin^4*^.

Correspondence to: Xihu Qin ([qinxihu@yeah.net](mailto:qinxihu@yeah.net)), Yuanxiang Lao ([yx_lao@126.com](mailto:yx_lao@126.com)) and Wenbin Yu ([wenbin_yu2003@163.com](mailto:sunbc@nju.edu.cn))

# These authors contributed equally to this work.

**This file includes:**

Supplementary Experimental Procedures

Supplementary Figures S1 to S10

Supplementary Tables S1 to S2

**Supplementary Experimental Procedures**

**Compounds and antibodies**

β-Hydroxybutyric acid (HY-113378), anagliptin ([HY-14877](https://www.medchemexpress.cn/Anagliptin.html)), erastin ([HY-15763](https://www.medchemexpress.cn/Erastin.html)), ferrostatin-1(HY-100579), Z-VAD-FMK (HY-16658B) and necrostatin-1s (HY-14622A) were purchased from MedChemExpress. Sodium Butyrate (S1539) was purchased from Beyotime. An antibody against acetyllysine (PTM-105) was purchased from PTM Biolabs. Antibodies against HMGCL (16898-1-AP), GAPDH (60004-1-lg), β-actin (20536-1-AP), KRT19 (10712-1-AP), NOX1 (17772-1-AP), EGFR (66455-1-Ig), H4 (16047-1-AP) and histone H3 (17168-1-AP) were purchased from Proteintech. H4ac (39026) were purchased from Activemotif. An antibody against DPP4 (YT5707) was purchased from Immunoway. Antibodies against LC3 (4599) and p62 (16177) were purchased from Cell Signaling Technology. Antibodies against p21 (ab107099), p16 (ab189034), HNF4α (ab181604), 4HNE (ab46545) and H3K9Ac (ab32129) were purchased from Abcam.

**Cell lines**

The human liver cancer cell lines Huh7, Hep3B, HepG2, MHCC-97L, MHCC-97H, HCC-LM3 and L02 were purchased from Shanghai Cell Bank of Chinese Academy of Science. The human kidney epithelial cell line HEK-293T was purchased from the American Type Culture Collection (ATCC). The majority of liver cancer cell lines were established from HCC patients. Huh7 was established from a highly differentiated tumor in the liver from a Japanese patient. MHCC-97H is a poorly differentiated cell line. All human liver cancer cells and HEK-293T cells were cultured in DMEM with 10% FBS, glutamine and penicillin–streptomycin (Wisent) at 37°C and 5% CO_2_. Mycoplasma contamination was excluded via a PCR-based method. The cell lines were authenticated by short-tandem-repeat DNA profiling.

**Clinical specimens**

Cohort 1 including HCC samples (n=252) obtained from patients who had undergone curative hepatectomy at Zhongshan Hospital, Fudan University (Shanghai, China) during 2007-2012. None of the patients in this study received radiotherapy or chemotherapy before surgery, and every patient was followed until June 2019. Follow-up procedures were described previously^1^. Briefly, patients were monitored by serum AFP, abdominal ultrasonography and chest radiography with an interval of 2-6 months according to the postoperative time. Enhancement computed tomography scanning (ECT) or magnetic resonance imaging (MRI) had been tested every 6-12 months or suspicious recurrence. Combined treatment modalities after recurrence were administered according to a uniform clinical guideline. Overall survival (OS) was defined as the interval between surgery and either death or the last observation taken. Tumor recurrence rate (RR) was defined as the rete of patients with HCC recurrence after opteration. If recurrence was not diagnosed, patients were censored on the date of death or the last follow-up. Clinical samples were collected from patients after obtaining informed consent in accordance with a protocol approved by the Ethics Committee of Zhongshan Hospital, Fudan University (Shanghai, China).

Cohort 2 including HCC samples including nonmetastatic HCCs (n=40, defined as NMH, without metastatic clinical manifestation and image features during 3-year follow-up after curative resection), metastatic HCCs (n=40, defined as MH, classic image features or pathologic diagnosis appeared within 1-year follow-up after curative resection), and paired adjacent normal liver tissues (n=80), which were obtained from the Affiliated Drum Tower Hospital of Nanjing University Medical School (Jiangsu, China). Clinical samples were collected from patients after obtaining informed consent in accordance with a protocol approved by the Ethics Committee of the Affiliated Drum Tower Hospital of Nanjing University Medical School (Jiangsu, China).

**Plasmids**

For HMGCL and DPP4 knockdown, the target shRNA sequence was subcloned into the pLVX-shRNA Lenti-vector. The shRNA sequences were as follows: shHMGCL: 5’- GCTGTCAGCACCTCATCTATG -3’; shDPP4:5’-CAGCAGCGUGAAUGAUAAA-3’. The CDS sequences of HMGCL and DPP4 were loaded into the pCDH-CMV plasmid to establish HMGCL and DPP4 overexpression plasmids. The primer sequence was as follow: HMGCL-F: 5'-GCCATGACACCTATGGTCAA-3'; HMGCL-R: 5'- CCATGACACCTATGGTCAA -3'.

For lentiviral infection, Huh7, Hep-3B, MHCC-LM3 and MHCC-97H were seeded in 6-well plates and cultured to 55-75% confluence. To generate lentivirus particles, HEK-293T cells were transfected with the above vectors (7.5μg) along with pSPAX2 (3.75μg) and pMD2.G (3.75μg) using lipofectamine® 3000 transfection reagent (Invitrogen) following the manufacturer’s. Puromycin was added 24h after transfection. Stable cells were cultured in a DMEM medium containing 10% FBS. shRES is a cell line of shHMGCL infected with a lentivirus overexpressing HMGCL. HMGCL and DPP4 related plasmids were purchased from Corues Biotechnology. All plasmid sequences are listed in Supplementary Table S1. Lentiviral knockdown and overexpression efficiency were assessed by western blot.

HMGCL and DPP4 overexpression were performed using pHB-CMV vectors obtained by cloning human HMGCL and DPP4 coding sequences. HMGCL ^(D42A, H233A)^ mutant lentiviral overexpression vectors were made by performing site-directed mutagenesis to introduce a sense mutation in their coding sequences.

**Lentivirus infection**

For lentivirus generation, 1×10^7^ HEK-293T cells were seeded in 10 cm dishes in DMEM supplemented with 10% FBS the day before transfection. Cells were transfected by changing to 10 mL of DMEM containing 15 μl Lipofectamine 3000 (Thermo Fisher Scientific, 11668027), 7.5 μg of pHB-U6-Luc shscrambled, pHB-U6-shHMGCL-Luc, pHB-CMV-Luc empty vector, pHB-CMV-HMGCL-Luc, pHB-CMV- HMGCL ^(D42A, H233A)^, combined with 3.75 μg psPAX2 and 3.75 μg pMD2G. After incubation overnight (at least 8 hours), the medium was changed to DMEM containing 10% FBS. After 48 hours, the supernatant was recovered, filtered with 0.45 mm filters and used to infect HCC cell lines. The stable cell lines were obtained by puromycin selection for 72 hours.

**Cell proliferation assay**

For the short-term proliferation assay, Huh7, Hep3B, HCC-LM3 and MHCC-97H cells were seeded into 96-well plates with 5,000 cell/well. CCK8 at a final concentration of 10% was added to each well and incubated for 1 h. The absorbance was measured at 450 nm. The data were analysed using GraphPad Prism 7 software.

For the long-term proliferation assay, cells were cultured and seeded into 6-well plates at a density of 1,000 cells per well, and were cultured in medium containing DMEM with 10% FBS for 14 days (medium was changed twice a week). Cells were fixed with 4% formaldehyde in PBS and stained with 0.1% crystal violet diluted in water. Cell confluence in each well was quantified using ImageJ software.

**Cell invasion and migration assays**

Transwell assay could be used to measure the invasion and migration ability of different cell lines. Cell invasion and migration were performed using a transwell plate (24-well insert, 8 μm pore size). Filters (Corning Inc., USA) were covered with 100 μl of matrigel (1:5 dilution; BD Biosciences). Then, 10×10^4^ cells were distributed in 100 μl of serum-free DMEM medium and inoculated into the upper chamber. Next, 600 μl of 90% DMEM medium supplemented with 10% FBS was added to the lower chamber. After 24 hours of incubation, the cultures were fixed with 4% paraformaldehyde for 30 minutes and then stained with 1% crystalline violet for 30 minutes. Finally, the number of invading and migrating cells under the upper chamber was counted under magnification.

**Wound healing assay**

Cells were incubated in 6-well plates at a concentration of 6×10^5^ cells/mL for 24 hours and then paddled in the 6-well plates with a 100 µL gun tip. After washing with PBS, DMEM medium without FBS was added to the plates. Photographs of the scratches were taken every 6 hours to calculate the proportion of the area healed from scratching.

**Quantitive RT-PCR**

Total RNA was extracted from normal liver tissue and HCC cell lines following the manufacturer's operation using the E.Z.N.A. Total RNA Kit I (Omega Bio-tek, GA, USA). The extracted total RNA needs to be stored in a -80°C refrigerator. The extracted RNA was reverse transcribed into complementary DNA (cDNA) with the use of HisScript Ⅱ (Vazyme, Shanghai, China). qRT-PCR was conducted on an ABI 7900 system using SYBR Green Ⅰ (Vazyme, Shanghai, China). All primers utilized in this investigation are listed in Table S2.

**Immunohistochemical staining**

IHC was performed according to our previous study^3^. Immunohistochemistry (IHC)

Paraffin tissue sections from 332 HCC patients and experimental animals were used for IHC staining. After the usual steps, tissues were stained with HMGCL (1:200, 16898-1-AP, Proteintech), DPP4 (1:200, YT5707, immunoway), Pan anti-acetyllysine (1: 200, PTM-105, Jingjie PTM BioLab) and H3K9ac (1: 200, ab32129, abcam) primary antibody for staining. The intensity of positive staining was defined as 1+, 2+, 3+ and 4+, indicating absent, weak, moderate and strong staining, respectively. The staining distribution was expressed as a percentage of positive tumor cells (0% to 100%). The two variables were multiplied together to obtain the final HMGCL expression score. Based on the HMGCL expression score, samples were further divided into low (1+, 2+) and high (3+, 4+) expression groups.

**ELISA**

Serum AFP concentration assays were performed using a mouse AFP ELISA kit (Nanjing Jiancheng Bioengineering Institute) according to theprotocols. Briefly, serum was added to a 96-well plate and coated with biotin-labelled antigen, and incubation was performed for 30 min at 37°C. After plate washing, avidin-HRP was added and incubated for 30 min at 37°C. Then, HRP chromogenic reagents A and B were added and incubated for 30 min at 37°C. After reaction termination, the visual color and absorbance of the resulting solution were qualitatively and quantitatively determined on a Tecan Infinite 200 PRO (TECAN). Analyses were performed in triplicate. The sigmoidal curves were calculated by mathematically fitting experimental points using Rodbard’s 4 parameter function. All measurements were conducted at room temperature.

**LC-MS/MS-based crotonylome analysis**

The metabolites were extracted from cell residue with 1 mL precooled methanol/acetonitrile/water (v/v, 2:2:1) under sonication for 1 h in ice baths. The mixture was incubated at -20 °C for 1 h followed by centrifugation at 14,000 g, 4 °C for 20 min, and then transferred to the sampling vial for LC-MS analysis.

Additionally, to ensure data quality for metabolic profiling, Quality control (QC) samples were prepared by pooling aliquots of all samples that were representative of the all samples under analysis, and used for data normalization. QC samples were prepared and analyzed with the same procedure as that for the experiment samples in each batch. Dried extracts were then dissolved in 50% acetonitrile. Each sample was filtered with a disposable 0.22 µm cellulose acetate and transferred into 2 mL HPLC vials and stored at -80°C until analysis.

Metabolomics profiling was analyzed using a UPLC-ESI-Q-TOF-MS system (UHPLC, 1290 Infinity LC, Agilent Technologies, Santa Clara, CA, USA) coupled TripleTOF 5600 (AB Sciex, Framingham, MA, USA).

For hydrophilic interaction liquid chromatography (HILIC) separation, samples were analyzed using a 2.1 mm × 100 mm ACQUIY UPLC BEH 1.7 μm column (Waters, Ireland). The flow rate was 0.5 mL/min and the mobile phase contained: A = 25 mM ammonium acetate and 25 mM ammonium hydroxide in water and B = acetonitrile (ACN). The gradient was 95% B for 0.5 min and was linearly reduced to 65% in 6.5 min, and then reduced to 40% in 2 min and maintained for 1 min, and then increased to 95% in 1.1 min, with 5 min re-equilibration period employed. Both electrospray ionization (ESI) positive-mode and negative mode were applied for MS data acquisition. The ESI source conditions were set as follows: Ion Source Gas 1 as 60, Ion Source Gas 2 as 60, curtain gas as 30, source temperature: 600 °C, IonSpray Voltage Floating (ISVF) ± 5500 V. In MS only acquisition, the instrument was set to acquire over the m/z range 60-1200 Da, and the accumulation time for TOF MS scanning was set at 0.15 s/spectra. In auto MS/MS acquisition, the instrument was set to acquire over the m/z range 25-1200 Da, and the accumulation time for product- ion scan was set at 0.03 s/spectra. The product-ion scan was acquired using information dependent acquisition with high sensitivity mode selected. The collisional energy was fixed at 30 V with ± 15 eV. Declustering potential was set as ± 60 V.

Quality control (QC) samples were prepared by pooling aliquots of all samples that were representative of the samples under analysis, and used for data normalization. Blank samples (75 %ACN in water) and QC samples were injected every six samples during acquisition.

**Bioinformatics analysis**

RNA-sequencing expression (level 3) profiles and corresponding clinical information for xx were downloaded from the TCGA dataset(https://portal.gdc.com). Ferroptosis-related genes from Ze-Xian Liu et al^2^. Systematic analysis of the aberrances and functional implications of ferroptosis in cancer.

The expression distribution of ferroptosis-related mRNA between HMGCL^high^ expression (n=93) and HMGCL^low^ expression (n=93) HCC tissues. The abscissa represents different ferroptosis, and the ordinate represents the expression distribution of gene, different colors represent different groups. *p < 0.05, **p < 0.01, ***p < 0.001, asterisks (*) stand for significance levels. The statistical difference of two groups was compared through the Wilcox test.

All the above analysis methods and R package were implemented by R foundation for statistical computing (2020) version 4.0.3.

**Immunoprecipitation**

Briefly, cellular lysates were prepared by incubating the cells in lysis buffer (50 mM Tris-HCl, 150 mM NaCl, 0.5% NP-40, and 2 mM EDTA, pH 7.5) containing protease and phosphatase inhibitor cocktail for 20 min at 4°C, followed by centrifugation at *14*,*000 × g* for 15 min at 4°C. The protein concentration of the lysates was determined using a BCA protein assay kit (Beyotime) according to the manufacturer’s protocol. Overall, 5% (1:20) cellular extracts were used for input. For immunoprecipitation, 500 μg of protein lysates was incubated with 2 μg of specific antibodies for 1 hour at 4°C with constant rotation; 20 μl of 50% protein A or G agarose beads was then added, and the incubation was continued overnight. The beads were then washed 4 times using lysis buffer. Between washes, the beads were collected by centrifugation at *500 × g* for 5 min at 4°C.

**Intercellular GSH assay**

The cells were washed 3 times with PBS. We collected the cells by centrifugation and added 3 times of the cell volume of the Protein Removal Reagent (M) solution. The samples were subjected to 2 times rapid freeze-thaws using liquid nitrogen and water at 37°C. Then, the samples were placed at 4°C or in an ice bath for 5 min. The samples were centrifuged at 10,000g for 10 min at 4°C. The supernatant was reserved for the determination of total glutathione. Using a 96-well plate, we added samples or standards in sequence and mix well. After adding 150 µl of total glutathione assay working solution, mix well and incubate at 25°C for 5 minutes. Then, the 50 µl of 0.5 mg/ml NADPH solution was added in the sample. Finally, the assay was determined using an enzymatic lab. Intracellular GSH levels were measured using a Total Glutathione Assay Kit (Beyotime, S0052). Results were normalized to the cell count.

**Intracellular cysteine assay**

Equilibrate all materials and prepared reagents to room temperature prior to use. We recommend that you make duplicate copies of all standards, controls and samples. Prepare a 1:10 dilution of Enzyme Preparation I. Add 2 μL of Enzyme Preparation I to 18 μL of Cysteine Assay Buffer. Mix enough reagent to cover the total number of wells to be assayed. Prepare 200µL of the reaction mixture for each reaction. Add 200µL of the reaction mixture to each reaction well. Then, we incubated the mix at 37°C for 30 minutes. The 30 μL enzyme mix Ⅱ was added in the each well and incubated at 37°C for 5 minutes. After incubation, we added 5 µL of cysteine probe to each well. Fluorescence needed to be measured at 365/450 nm in kinetic mode for at least 30 minutes at room temperature. Intracellular cysteine levels were measured using a cysteine assay (abcam, NO: ab211099).

**Detection of iron concentration**

Iron concentrations were analysed using an iron detection kit (Sigma, NO:MAK025). Briefly, cells were grown to mid-log phase, washed, suspended in PBS and homogenized using 3 times freeze and thaw cycle. 5 μL of iron assay buffer was mixed with 100 μL cell free extract. The mixture was incubated at 25°C for 30 minutes. 100μL of iron probe was added to the mixture and was incubated at room temperature for additional 60min. Fluorescence needed to be measured at 593nm.

**Detection of MDA**

Use 0.1 ml of lysate or homogenate per 1×106 cells. After lysate or homogenate, cells were centrifuged at 10,000g-12,000g for 10 minutes. Then, we saved the supernatant for subsequent assays. We used 0.1 ml of sample for the tested and utilized 0.1 ml of PBS to the centrifuge tube as a blank control. The mixture was mixed well and heated at 100°C for 15 minutes. The sample and blank control was cooled to room temperature and centrifuged at 1000g for 10 minutes at room temperature. 200 µl of supernatant was added to a 96-well plate and the absorbance was subsequently measured at 532 nm using an enzymatic lab. MDA was mearsured utilizing Lipid Peroxidation MDA Assay Kit (Beyotime, NO: S0131).

**Half-maximal inhibitory concentration assay (IC50)**

The cells were seeded in 96-well plates with 1× 104 cells per well. The corresponding concentrations of sorafenib were given to cells for 24 h. After 24 h, CCK-8 (MCE, NO: HY-K0301) was used to measure sorafenib sensitivity at 450 nm using a microplate reader (Thermo Fisher, USA) after incubating for 1 h at 37 °C.

**CUT＆Tag assay**

The Hyperactive In-Situ ChIP Library Prep Kit (pG-Tn5) (Vazyme Biotech Co., Ltd#TD901-01) was used to assess the DNA binding of HMGCL-H3K9ac. The CUT＆Tag was performed with an anti-H3K9ac antibody (1: 50, ab32129, abcam). Goat anti-rabbit IgG secondary antibody (ABin101961) was utilized at 1:100 dilution. The library was prepared using Huh7 shNT and Huh7 shHMGCL cells. Briefly, Huh7 shNT and Huh7 shHMGCL cells were cultured in DMEM medium and approximately 1×105 cells were used for the library. Cells were washed in pre-cooled PBS and resuspended in pre-cooled lysis buffer (10 mM Tris-HCl, pH 7.4, 10 mM NaCl, 3 mM MgCl2, 0.1% NP-40). Labelling reactions were performed using Tn5 transposase (TD501; Vazyme, China). After obtaining purified DNA, amplification and barcoding were performed with NEBNext High Fidelity 2× PCR Master Mix (New England Biolabs). DNA fragments were purified using AMPure beads (Beckman Coulter, Brea, CA, USA) and library quality was analysed using a BioAnalyzer 2100. Sequencing was performed using a HiSeq X Ten sequencer (Illumina, supplied by Novogene, China).

**Electron microscopy**

Cells from the CONTROL and HMGCL groups were immersed in the fixative 2.5% glutaraldehyde and stored at 4°C. Ultrastructural images were captured using a transmission electron microscope (Hitachi HT7700, Japan).

**Membrane and nuclear protein extraction**

Membrane and nuclear protein were extracted based on the operation instruction of Oproteome Cell Compartment Kit (Beyotime, No. P0028).

**NADPH oxidase assay**

The NADPH oxidase (NOX) activity was count using colorimetric method. First, cells were collected in lysis buffer, and centrifuged at 600 x g for 3 minutes at 4°C. The supernatant (100 µL) was taken and transferred to a 96-well plate. NADPH (250 µmol/L) was added and the absorbance was assessed at 340 nm. After incubation at room temperature for 5 minutes, the absorbance was assessed again.

**Senescence β-Galactosidase Staining**

Huh7 and MHCC-LM3 cells were utilized for the β-galactosidase assay by adding 1 ml of β-galactosidase staining fixative and fixing at room temperature for 15 minutes. Staining was performed using 1 ml of staining working solution, and the staining working solution was configured according to the instructions of Senescence β-Galactosidase Staining Kit (Beyotime, No:C0602).

**Data Availability**

Raw and processed data from crotonylome analysis of samples will be deposited in the ProteomeXchange Dataset. All other data will be found in the source data, supplementary information is available from the corresponding authors upon reasonable request.

**References**

1. Lu M, Zhu WW, Wang X, Tang JJ, Zhang KL, Yu GY, et al. ACOT12-Dependent Alteration of Acetyl-CoA Drives Hepatocellular Carcinoma Metastasis by Epigenetic Induction of Epithelial-Mesenchymal Transition. Cell Metab 2019; 29:886-900.e5.

2. [Liu Z, Zhao Q, Zuo ZX, Yuan SQ, Yu K, Zhang Q, Zhang X, Sheng H, Ju HQ, Cheng H, Wang F, Xu RH, Liu ZX. Systematic Analysis of the Aberrances and Functional Implications of Ferroptosis in Cancer. iScience. 2020 Jul 24;23(7):101302.](https://www.ncbi.nlm.nih.gov/pmc/articles/PMC7334617/)

3. Postic C, Magnuson MA. DNA excision in liver by an albumin-Cre transgene occurs progressively with age. Genesis 2000; 26:149-150.

**Supplemental Figures**


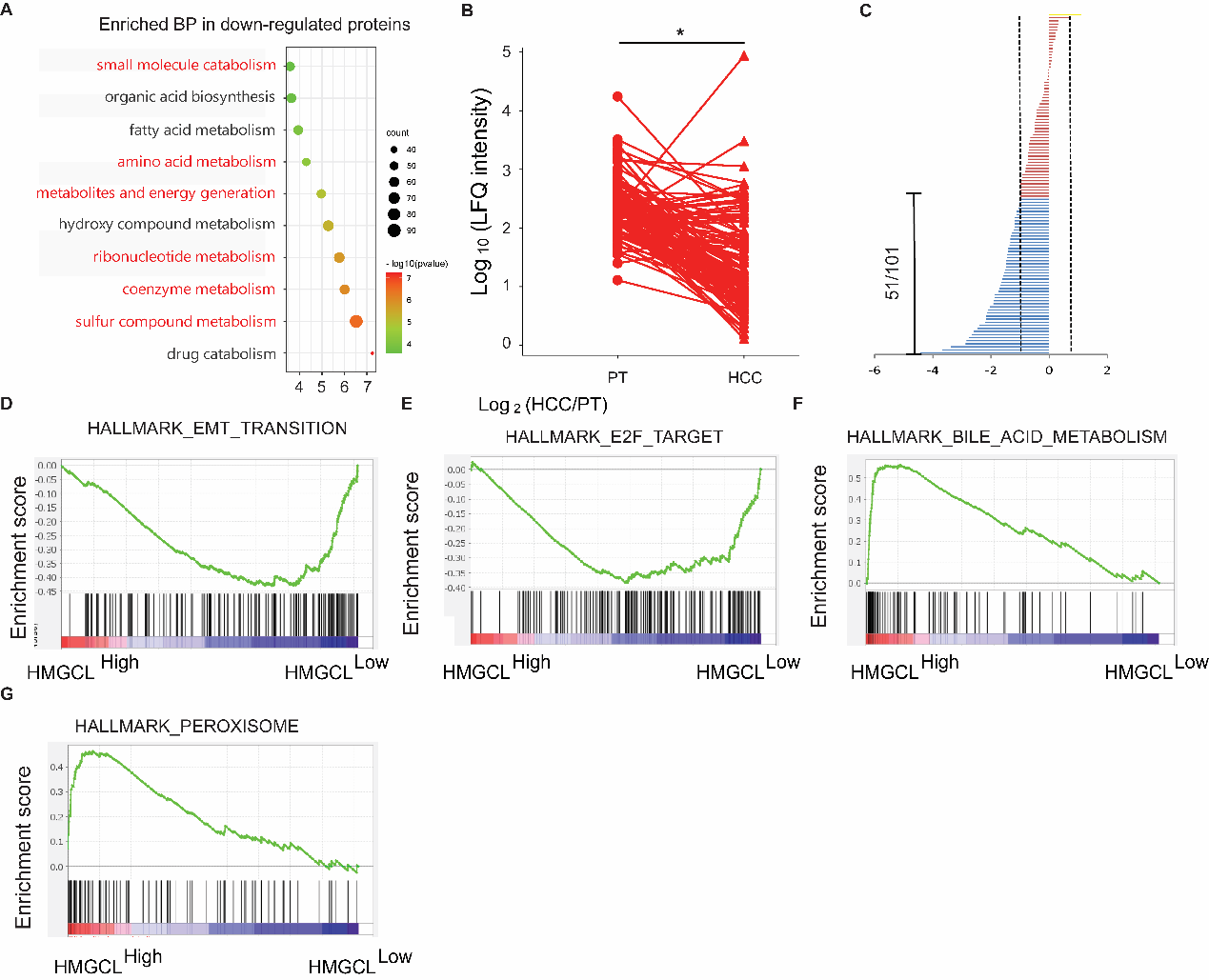


**Supplement figure 1: Protein abundance of HMGCL was downregulated in HCC tissue and was involved in a variety of metabolic processes.** (A, B): Protein abundance of HMGCL in HCC tissue was presented used scatterplot and histogram (n=101). Public Data was share by JIANG. (C-G): Metabolic and molecular pathways activated by downregulation of HMGCL. *P < 0.05, **P < 0.01, ***P < 0.001, ns as no significance. (B) Paired t-test.


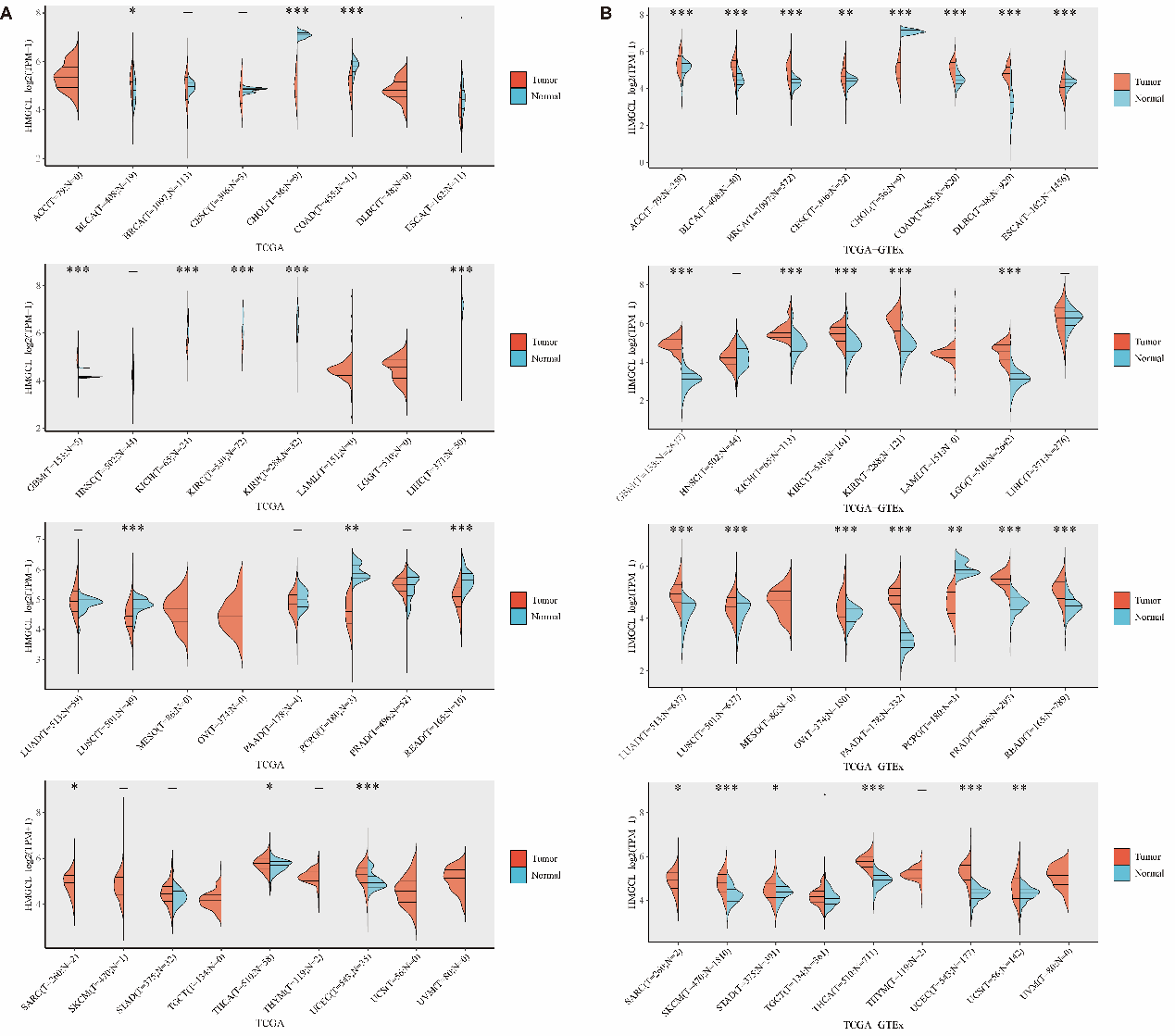


**Supplement figure 2: The expression pf HMGCL in pan-cancer.** (A): The expression of HMGCL in pan-cancer downloaded from the TCGA database. (B): The expression of HMGCL in pan-cancer downloaded from the TCGA + GTEx database. *P < 0.05, **P < 0.01, ***P < 0.001. (A, B) Wilcox test.


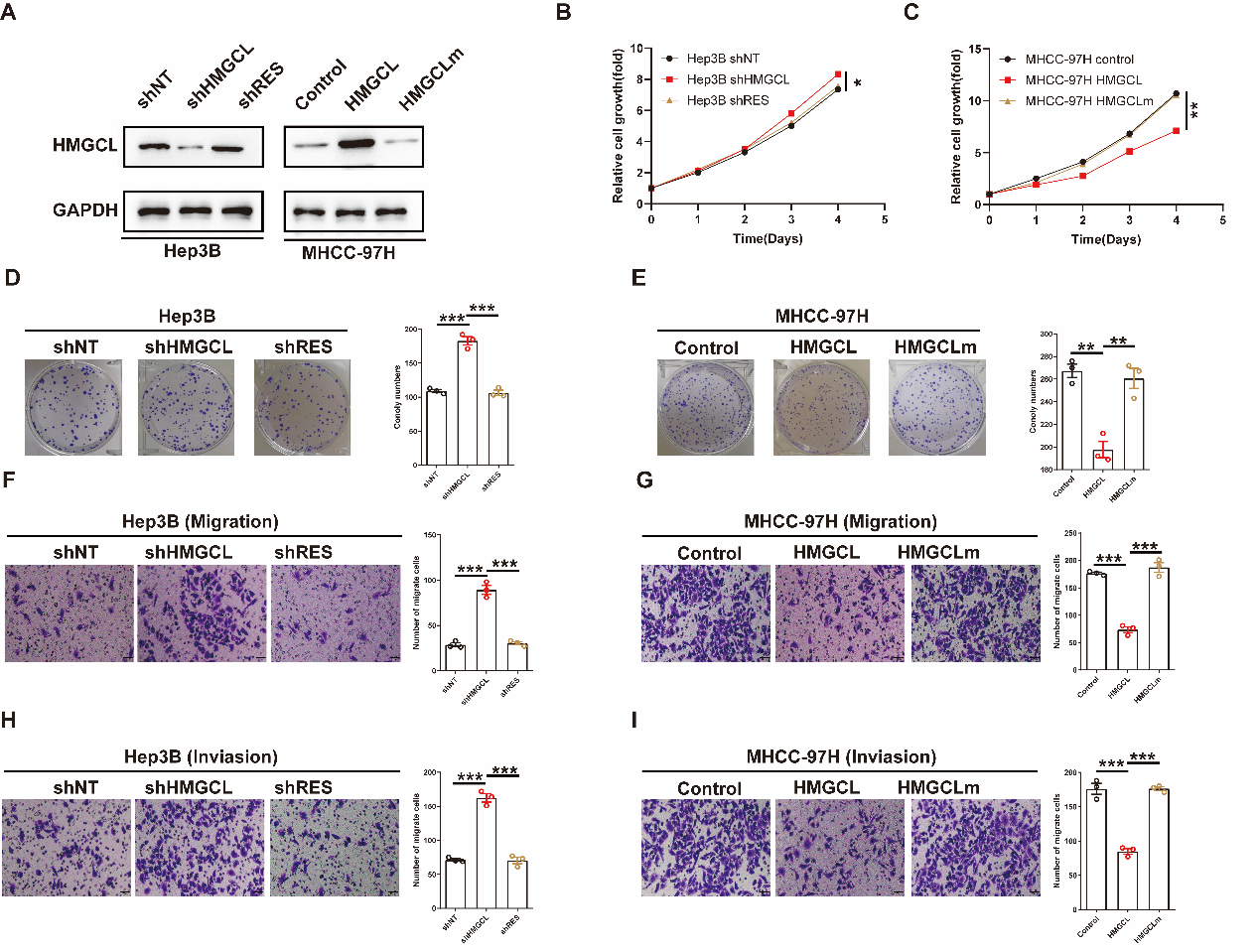


**Supplement figure 3: HMGCL suppresses HCC metastasis and proliferation *in vitro*.** (A) Confirmation of HMGCL knockdown (KD, shHMGCL), re-expression (shRES), overexpression (HMGCL) and overexpression with mutation site (HMGCLm) in HCC cell lines. (B, C) The effect of HMGCL gain- or loss-of-function on *in vitro* proliferation utilized CCK8 assay. The relative cell number was counted as fold change to Day 0. (D, E) The effect of HMGCL gain- or loss-of function on *in vitro* proliferation utilized colony formation assay. Cell numbers was counted at day 14. (F-I) Used transwell assay measured the effect of HMGCL gain- or loss-of-function of *in vitro* migrated and invaded cells numbers. Each experiment was performed at least three times, all data was showed as mean ± SD. *P < 0.05, **P < 0.01, ***P < 0.001, ns as no significance. (B-I) Mean ± SD; One-way ANOVA test.


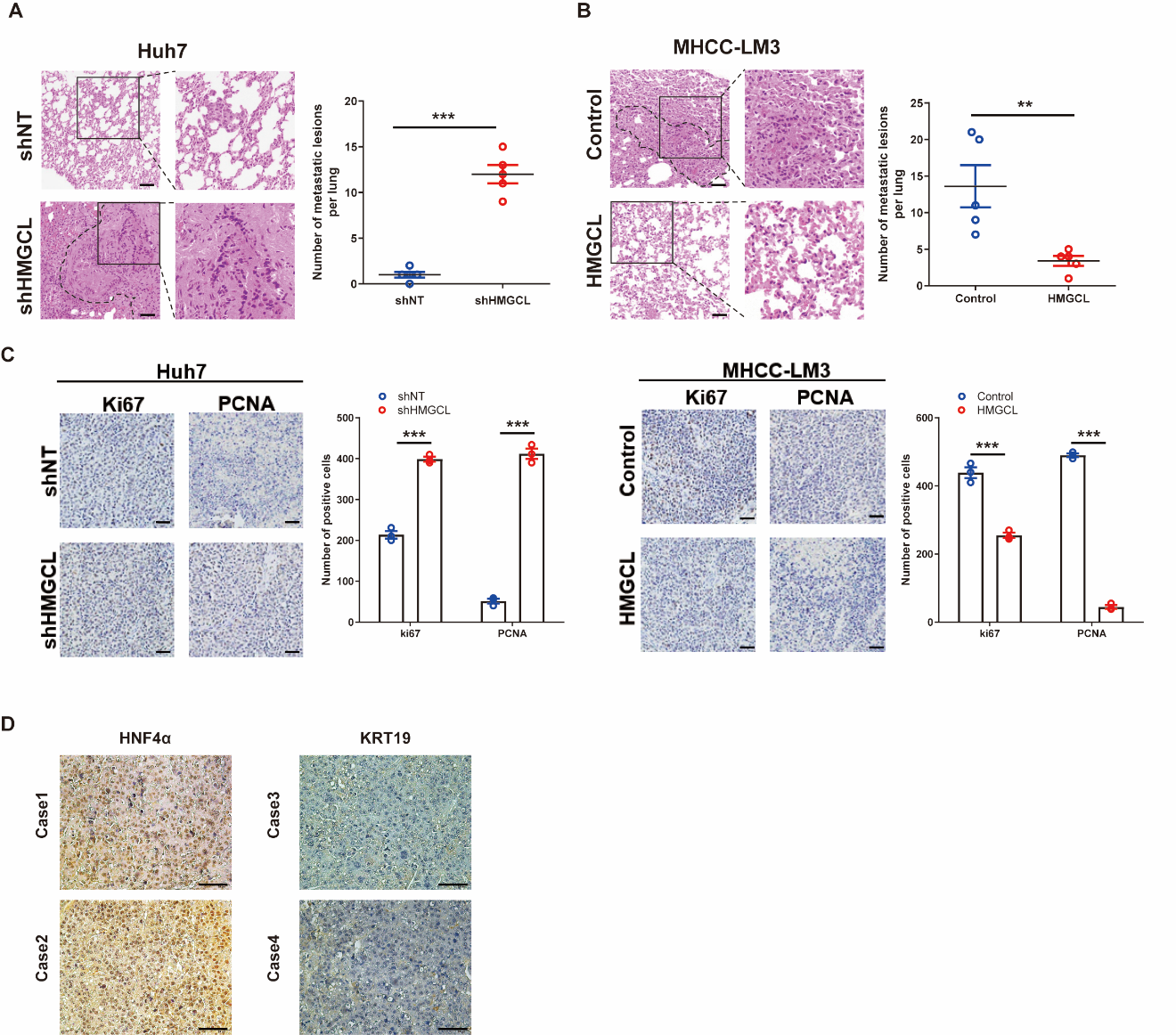


**Supplement figure 4: HMGCL suppresses HCC metastasis and proliferation *in vivo*.** (A, B) The lung metastasis model was established in nude mice by tail vein injection of Huh7 cells or MHCC-97H cells. (C) Ki67 and PCNA staining were performed in xenograft, scare bar: 100μm, positive cells number was presented in the column chart. (D) Trp53^Δhep/Δhep^ driven tumors were staining with HNF4α and KRT19. Each experiment was performed at least three times, all data was showed as mean ± SD. *P < 0.05, **P < 0.01, ***P < 0.001, ns as no significance.


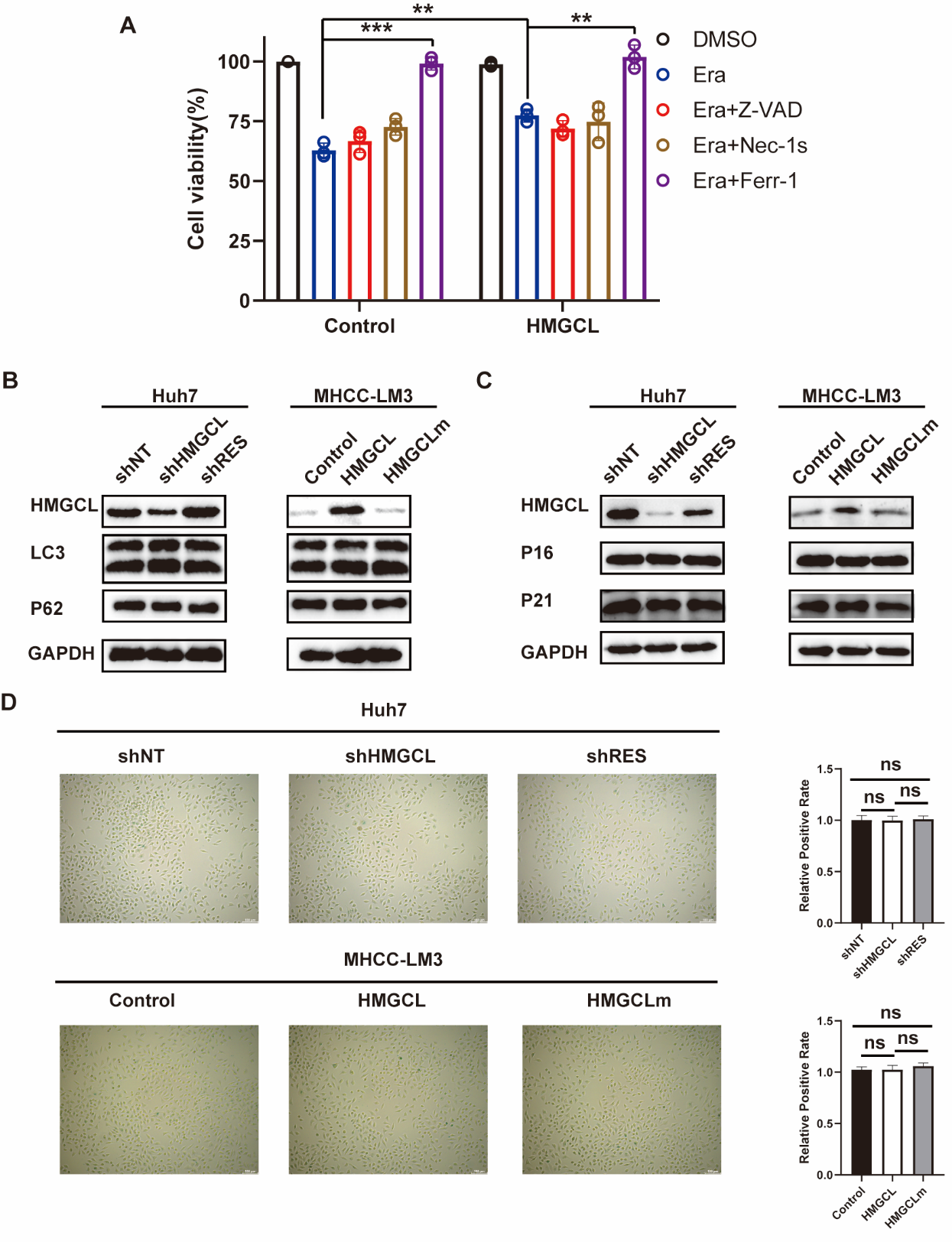


**Supplement figure 5: Expression level of HMGCL was associated with ferroptosis and not related with autophagy and senescence.** (A) Overexpression of HMGCL prompted the ferroptosis sensitive of HCC cells. (B) The relationship between regulation the expression of HMGCL and autophagy. (C) The relationship between regulation the expression of HMGCL and senescence. (D) Imaging of β-galactosidase staining positivity by altered expression of HMGCL. Each experiment was performed at least three times, all data was showed as mean ± SD. *P < 0.05, **P < 0.01, ***P < 0.001, ns as no significance. (C) Mean ± SD; One-way ANOVA test.


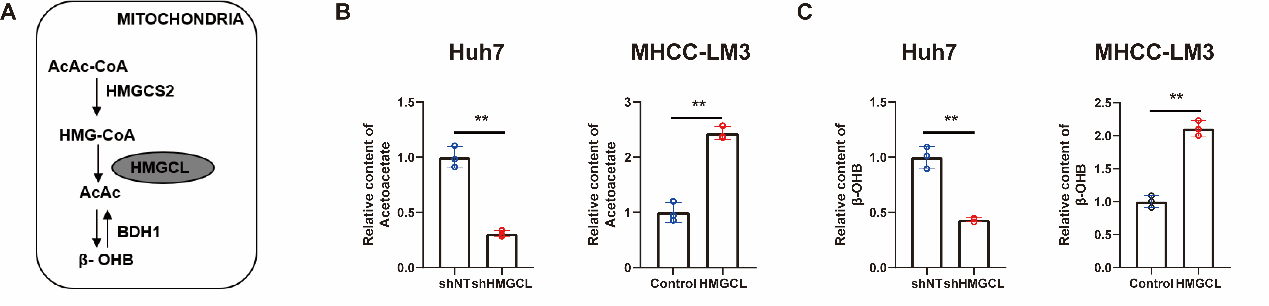


**Supplement figure 6: The role of HMGCL in ketone body production.** (A) The diagram showed the role of HMGCL in the process of β-OHB production. (B) Effect of altered the expression of HMGCL on acetoacetate. (C) Effect of altered the expression of HMGCL on β-OHB. Each experiment was performed at least three times, all data was showed as mean ± SD. *P < 0.05, **P < 0.01, ***P < 0.001, ns as no significance. (B-C) Mean ± SD; One-way ANOVA test.


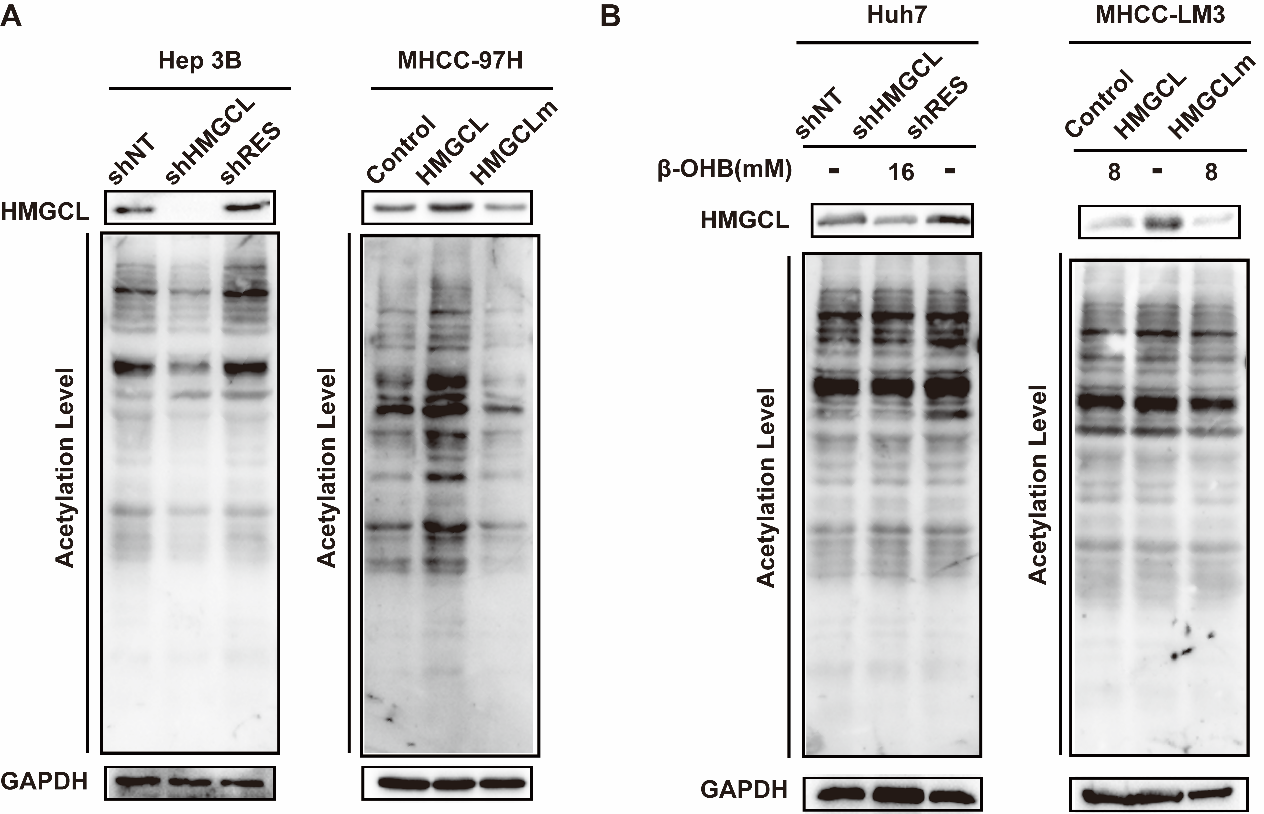


**Supplement figure 7:** **HMGCL affects the total acetylation level of the protein.** (A) HMGCL affects the total acetylation level of total proteins extracted from Hep3B cells and MHCC-97H cells. (B) Effect of adding exogenous β-OHB on the total acetylation level of total protein extracted from Huh7 and MHCC-LM3 cells with different HMGCL expression.


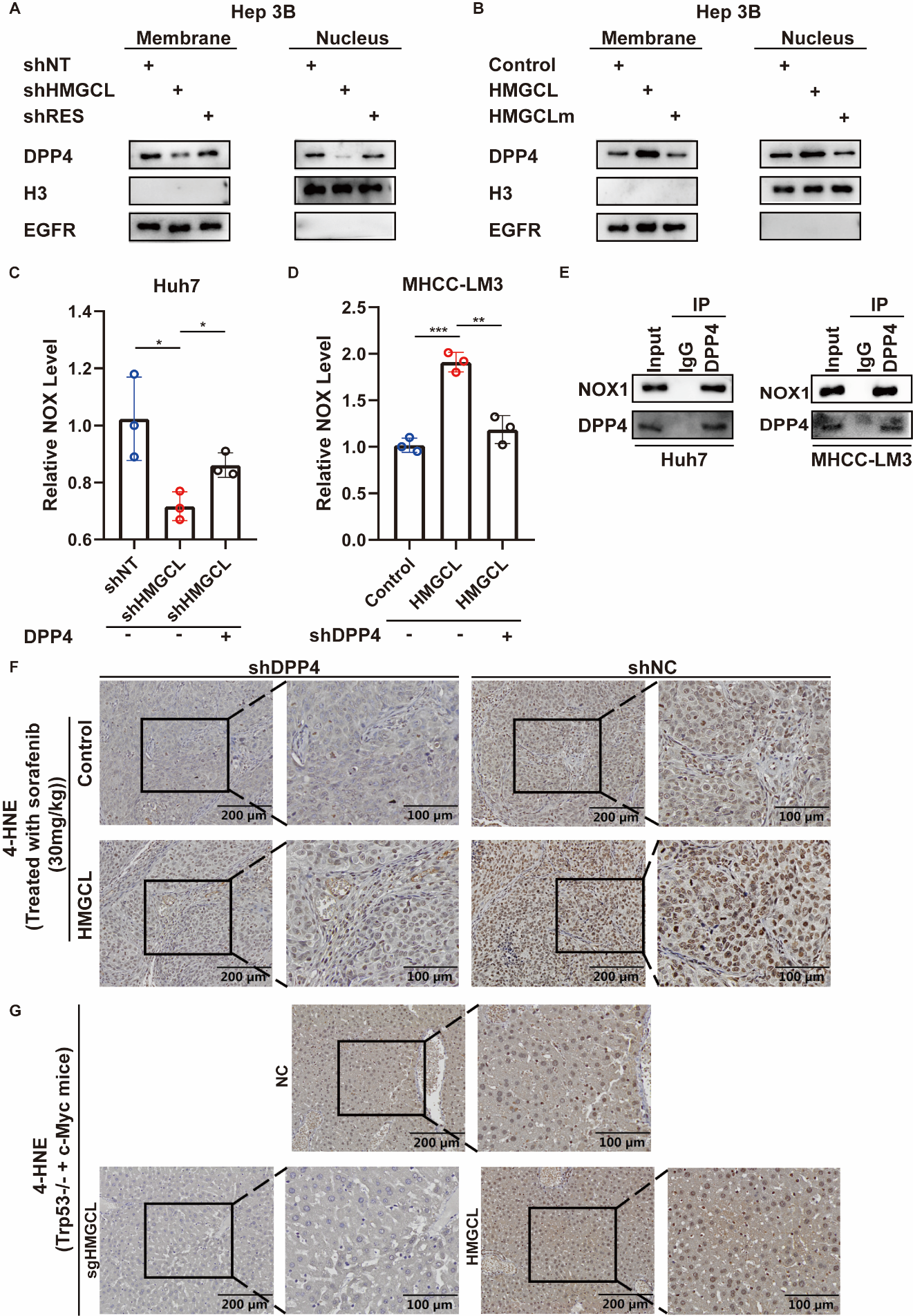


**Supplement figure 8: The cell localization of DPP4 and its relation with NOX activity, and the 4-HNE expression in nude mice and *Trp53-/-; c-Myc* mice.** (A, B) Cell localization of altered DPP4 in HMGCL changing Huh7 and MHCC-LM3 cells. (C, D) Effect of changing expression of HMGCL and DPP4 on NOX activity in Huh7 and MHCC-LM3 cells. (E) The ability to bind to each other between DPP4 and NOX1. (F) DPP4 deletion impaired the expression of 4-HNE in HMGCL^OE^ nude mice. (G) The alteration of HMGCL in *Trp53-/-; c-Myc* mice could change the expression of 4-HNE. Each experiment was performed at least three times, all data was showed as mean ± SD. *P < 0.05, **P < 0.01, ***P < 0.001, ns as no significance. (C, D) Mean ± SD; One-way ANOVA test.


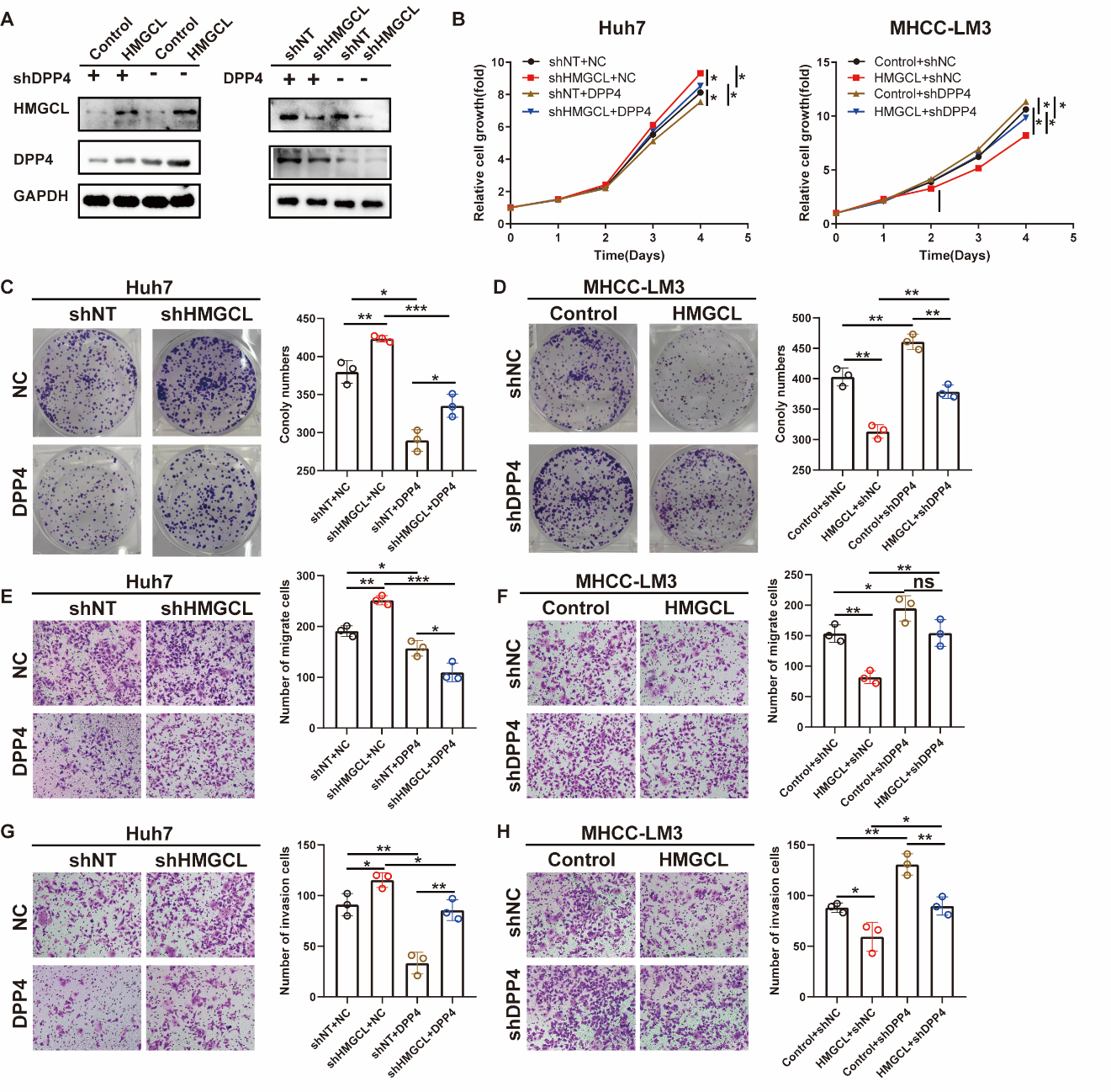


**Supplement figure 9: DPP4 suppresses the malignant phenotype of HCC affected by altered HMGCL expression.** (A) shNT and shHMGCL cells were transfected with DPP4 and NC. Control and HMGCL cells were transfected with shDPP4 and shNC. (B-D) Effect of altered DPP4 expression on the proliferation of HMGCL and shHMGCL cells. (E-H) Effect of altered DPP4 expression on migration and invasion of HMGCL and shHMGCL cells. Each experiment was performed at least three times, all data was showed as mean ± SD. *P < 0.05, **P < 0.01, ***P < 0.001, ns as no significance. (B-H) Mean ± SD; One-way ANOVA test.


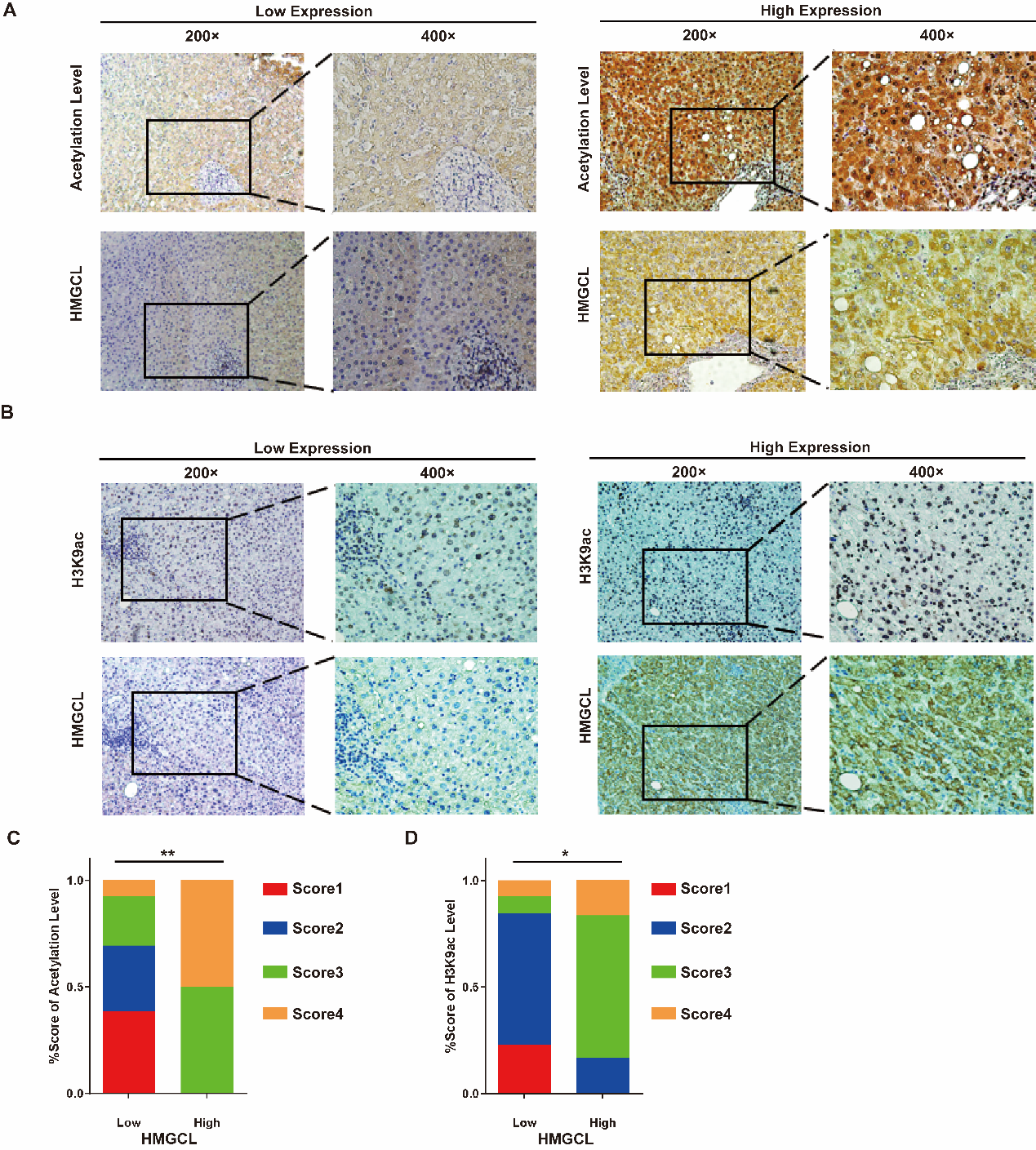


**Supplement figure 10:** HMGCL affects the total acetylation level of the protein in HCC tissues. (A, C) The correlation between the expression of HMGCL and acetylation levels using IHC assay (including 25 cases of HCC samples with high expression of HMGCL and 25 cases of HCC samples with low expression of HMGCL). The results of the analysis are shown using bar charts. (B, D) The correlation between the expression of HMGCL and the expression of H3K9ac using IHC assay (including 25 cases of HCC samples with high expression of HMGCL and 25 cases of HCC samples with low expression of HMGCL). The results of the analysis are shown using bar charts. Each experiment was performed at least three times, all data was showed as mean ± SD. *P < 0.05, **P < 0.01, ***P < 0.001, ns as no significance. (C, D) Significance was determined using the λ2 test. Data are shown as percentage of total specimen.

**Supplemental Table1**

| **Table S1: Plasmid sequences used in this research (5’-3’)** | |
| --- | --- |
| Name | Sequence |
| HMGCL shRNA | 5′- GCTGTCAGCACCTCATCTATG -3′ |
| DPP4 shRNA | 5′- CAGCAGCGUGAAUGAUAAA -3′ |
| shNT | 5′- UUCUCCGAACGUGUCACGU -3′ |
| shNC | 5′- UUCUCCGAACGUGUCACGUTT -3′ |
| sgHMGCL | 5′- GATCAGCCTGATCTTCACTG -3′ |

**Supplemental Table2: Primers sequences used in this research (5’-3’)**

| Name | Sequence |
| --- | --- |
| HMGCL forward | 5′- CACAGCGAGGTTTTGCTCAG-3′ |
| HMGCL reverse | 5′- AACTGGGTAGGGACGAGGAG-3′ |
| HSPA5 forward | 5′-CCGAGGAGGAGGACAAGAAGGAG-3′ |
| HSPA5 reverse | 5′-TTGGCGTCAAAGACCGTGTTCTC-3′ |
| EMC2 forward | 5′- GGCGAAGGTCTCAGAGCTTTACG-3′ |
| EMC2 reverse | 5′- CAATGCCAAGTCATCCCGACCATAG-3′ |
| MT1G forward | 5’- CTTCTCGCTTGGGAACTCTA -3’ |
| MT1G reverse | 5’- AGGGGTCAAGATTGTAGCAAA -3’ |
| HSPB1 forward | 5′- GGCGAAGGTCTCAGAGCTTTACG-3′ |
| HSPB1 reverse | 5′- CAATGCCAAGTCATCCCGACCATAG-3′ |
| FANCD2 forward | 5′- CTCAGCCAGAGCGTCCATTACTTG-3′ |
| FANCD2 reverse | 5′-GACACCAACACCAGCAATCTCCTC-3′ |
| SLC1A5 forward | 5′- CTGATGATGAAGTGCGTGGAGGAG-3′ |
| SLC1A5 reverse | 5′-GGTTGACTGCTTCGAGGATGATGG-3′ |
| TFRC forward | 5′- GCTGTATTCTGCTCGTGGAGACTTC-3′ |
| TFRC reverse | 5′-CGTCACCAGAGAGGGCATTTGC-3′ |
| RPL8 forward | 5′- CACGGCTACATCAAGGGCATCG-3′ |
| RPL8 reverse | 5′- GTTGTGGGAGATAACGGTGGCATAG-3′ |
| LPCAT3 forward | 5′- AAAGGGCAAGGCAAAGTGGGATG-3′ |
| LPCAT3 reverse | 5′- GCAGAAGGCAGTCATGGAGTAACC-3′ |
| DPP4 forward | 5′- AGTGGCGTGTTCAAGTGTGGAATAG-3′ |
| DPP4 reverse | 5′- ATGTTGGTGTGCTGTGCTGCTAG-3′ |
| CS forward | 5′- CTACAGAGAAGGCAGCGGTATTGG-3′ |
| CS reverse | 5′- CAAAGGACAGGTAAGGGTCGGAAAG-3′ |
| CARS1 forward | 5′- GTGTGGAGGTGTTGCTGGAAGAAG-3′ |
| CARS1 reverse | 5′- ATTGGAGACATAGCCGTAACCGTTG-3′ |
| ACSL4 forward | 5′- TGGGCTAAATGAATCTGAGGCTTCC-3′ |
| ACSL4 reverse | 5′- GGCGTTGGTCTACTTGGAGGAATG-3′ |
| GPX4 forward | 5′- CCCGATACGCTGAGTGTGGTTTG-3′ |
| GPX4 reverse | 5′- CCTTGCCCTTGGGTTGGATCTTC-3′ |
| GAPDH forward | 5′-AAGGTGAAGGTCGGAGTCAAC-3′ |
| GAPDH reverse | 5′-GGGGTCATTGATGGCAACAATA-3′ |
